# Antisense Oligonucleotide-Based Rescue of Aberrant Splicing Defects Caused by 15 Pathogenic Variants in *ABCA4*

**DOI:** 10.3390/ijms22094621

**Published:** 2021-04-28

**Authors:** Tomasz Z. Tomkiewicz, Nuria Suárez-Herrera, Frans P. M. Cremers, Rob W. J. Collin, Alejandro Garanto

**Affiliations:** 1 Department of Human Genetics and Donders Institute for Brain, Cognition and Behaviour, Radboud University Medical Center, 6525GA Nijmegen, The Netherlands; tomasz.tomkiewicz@radboudumc.nl (T.Z.T.); nuria.suarezherrera@radboudumc.nl (N.S.-H.); frans.cremers@radboudumc.nl (F.P.M.C.); rob.collin@radboudumc.nl (R.W.J.C.); 2 Departments of Pediatrics and Human Genetics, Radboud Institute for Molecular Life Sciences, Radboud University Medical Center, 6525GA Nijmegen, The Netherlands

**Keywords:** antisense oligonucleotide, *ABCA4*, Stargardt disease, inherited retinal diseases, splicing modulation, RNA therapy, deep-intronic, near-exon, pseudoexon, exon elongation

## Abstract

The discovery of novel intronic variants in the *ABCA4* locus has contributed significantly to solving the missing heritability in Stargardt disease (STGD1). The increasing number of variants affecting pre-mRNA splicing makes *ABCA4* a suitable candidate for antisense oligonucleotide (AON)-based splicing modulation therapies. In this study, AON-based splicing modulation was assessed for 15 recently described intronic variants (three near-exon and 12 deep-intronic variants). In total, 26 AONs were designed and tested in vitro using a midigene-based splice system. Overall, partial or complete splicing correction was observed for two variants causing exon elongation and all variants causing pseudoexon inclusion. Together, our results confirm the high potential of AONs for the development of future RNA therapies to correct splicing defects causing STGD1.

## 1. Introduction

Stargardt disease (STGD1, OMIM: 248200) is characterized by progressive degeneration of the retina. Although STGD1 is usually considered a juvenile disease, the age of onset depends on the severity of the combination of variant(s) [1,2,3,4,5,6]. STGD1 patients can experience a diverse range of clinical manifestations from severe visual impairment to complete blindness. The three main clinical features include progressive degeneration of the central macula, presence of autofluorescent lipofuscin flecks distributed across the macula, and surprising preservation of the tissue surrounding the optic nerve [7].

STGD1 is caused by biallelic mutations in the *ABCA4* locus. The 50-exon gene encodes the ATP-binding cassette subfamily A member 4 (ABCA4) protein, which plays a crucial role in photoreceptor cell maintenance. ABCA4 is a transmembrane protein located in the rims of the outer segment discs in rods and lamellae in cones [8]. It functions as a flippase transporter and facilitates the removal of potentially toxic retinoic acid derivatives generated during the visual cycle from the lumen to the photoreceptor cell cytosol [9]. Therefore, ABCA4 dysfunction drives the accumulation of the toxic derivatives and accelerates the formation of toxic bisretinoids compounds such as *N*-retinylidene-*N*-retinyl-phosphatidyl-ethanolamine (A2PE) in the photoreceptor outer segment. A2PE is hydrolyzed to *N*-retinylidene-*N*-retinyl-ethanolamine (A2E) upon phagocytosis of photoreceptor outer segments by the retinal pigmented epithelium (RPE) cells. This increases the oxidative damage triggered by the A2E as part of lipofuscin accumulation in the RPE cell, causing cellular damage and consequently, photoreceptor cell death and progressive loss of vision [10,11,12].

The phenotypic and allelic heterogeneity of STGD1 complicates a proper genetic diagnosis. In the past, genetic testing was focused on the coding regions of a gene. In recent years, next-generation sequencing has allowed the sequencing of exons and introns, which has led to new insights. For instance, in 448 STGD1 cases found to carry two *ABCA4* alleles after next-generation sequencing, 11% and 10% of the causal alleles consisted of deep-intronic and non-canonical splice site (NCSS) variants, respectively [13]. The combination of in-depth *ABCA4* sequencing, in silico evaluation using splice prediction tools and midigene-based splice assays, has contributed to solving the missing heritability present in STGD1. So far, over 1200 STGD1-causing variants in *ABCA4* have been reported (https://databases.lovd.nl/shared/genes/ABCA4, last accessed day 22 April 2021) [14,15,16,17,18,19,20]. Pathogenic intronic and NCSS variants can result in aberrant splicing. Such variants are excellent candidates for antisense oligonucleotide (AON)-based splicing modulation therapeutic strategies. Our group and others have previously demonstrated the great potential of AONs to rescue the effect of mutations disrupting the splicing process in several inherited retinal disease-associated genes, i.e., *CEP290* [21,22,23,24,25,26], *USH2A* [27], *CHM* [28], *OPN1* [29] and *ABCA4* [18,30,31,32]. 

Recently, 15 novel *ABCA4* intronic variants have been reported [13,17,31,33]. Three variants were classified as near-exon variants and 12 as deep-intronic variants. In the case of the first group, variants c.1937+37C>G, c.3191-11T>A and c.4352+61G>A have been described to cause an exon elongation in the final transcript [13,17,31]. In contrast, the 12 deep-intronic variants alter the pre-mRNA splicing by inserting a pseudoexon (PE) into the final mRNA, causing a frameshift and premature termination of translation.

Here, we report the design of a set of AONs able to redirect the splicing defects caused by these 15 recently described *ABCA4* intronic variants, thereby providing new opportunities for further therapeutic development of STGD1.

## 2. Results

### 2.1. Design of Antisense Oligonucleotides

**Figure 1:**
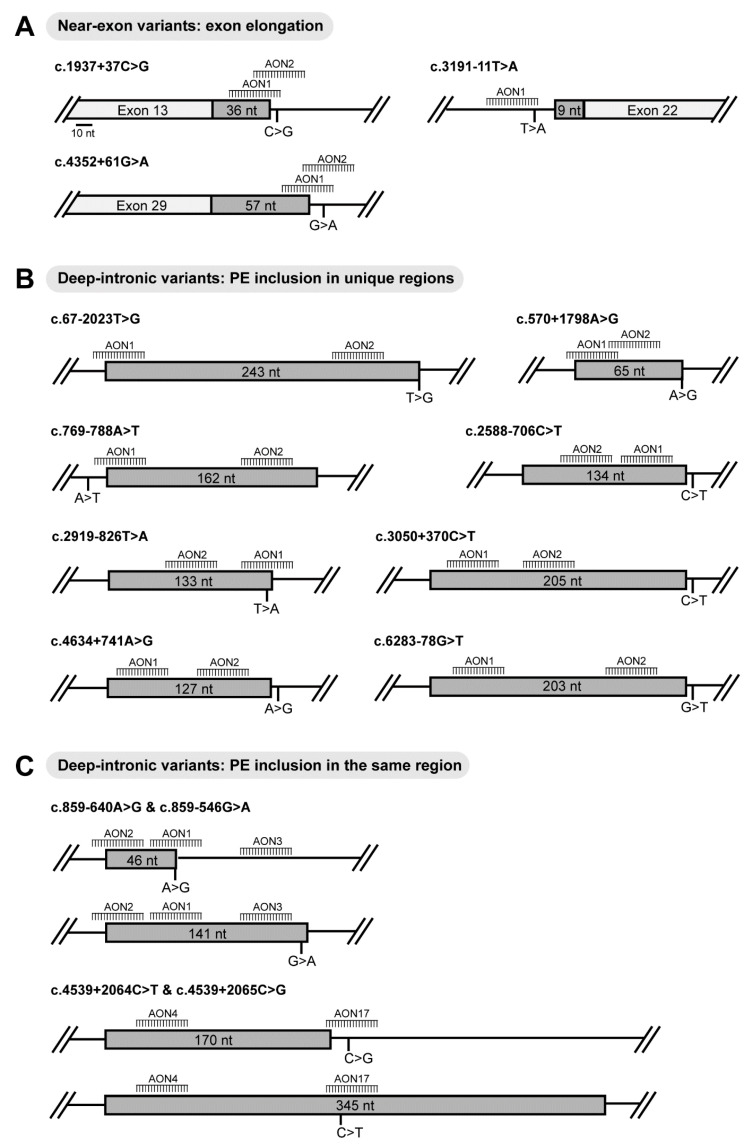
Schematic representation of the different splicing alterations caused by 15 intronic *ABCA4* variants and the respective position of the designed 26 antisense oligonucleotides (AONs) in this study. (**A**) Position of the AONs targeting exon elongation caused by the indicated near-exon variants. (**B**) Position of the AONs targeting the corresponding pseudoexon (PE) inclusion caused by the indicated deep-intronic variants in unique regions. (**C**) Position of the AONs targeting the corresponding pseudoexon (PE) inclusion caused by the indicated deep-intronic variants in the same region.

Fifteen variants disrupting *ABCA4* splicing were selected for rescue studies using AONs. All variants were confirmed as STGD1-causing variants and their effect at RNA level was validated by in vitro splice assays using midigenes [13,17,19,31,33,34,35] (Supplementary Table S1). A total of 26 AONs and one scramble-oligonucleotide (SON) modified with 2′-methoxyethyl and phosphorothioate (2′-*O*-MOE-PS) were tested in the midigenes harboring one of the 15 corresponding variants (Table 1). From the 26 AONs, 24 were newly designed and the last 2 AONs (AON4 and AON17) were from a previous study for c.4539+2001G>A and c.4539+2028C>T variants [30,32]. They were used in this study to assess their splicing rescue efficacy for the c.4539+2064C>T and c.4539+2065C>G splicing defects. For the near-exonic variants, the AONs were designed to block the aberrant splice site that was created by each variant, preventing accessibility of the spliceosome. For the deep-intronic variants, the best strategy involved targeting strong exonic splicing enhancer (ESE) motifs present within the PEs (Figure 1, Supplementary Figure S1) [36]. 

Two AONs were used for each variant, except for c.3191-11T>A, c.859-640A>G and c.859-546G>A. The c.3191-11T>A variant is located 11 nt upstream of exon 22. The variant is in close proximity to *cis*-acting splice regulatory features such as the splicing acceptor site (SAS), the branch point, and high-density splice enhancer motifs. The presence of crucial splice governing features allowed the design of only a single AON for this variant (Figure 1A). The c.859-640A>G and c.859-546G>A variants caused overlapping PEs; 46 nt and 141 nt in size, respectively. Three AONs were designed to eliminate these PEs in intron 7 (Figure 1C).

### 2.2. AON-Driven Splicing Modulation for Near-Exon Variants

**Figure 2:**
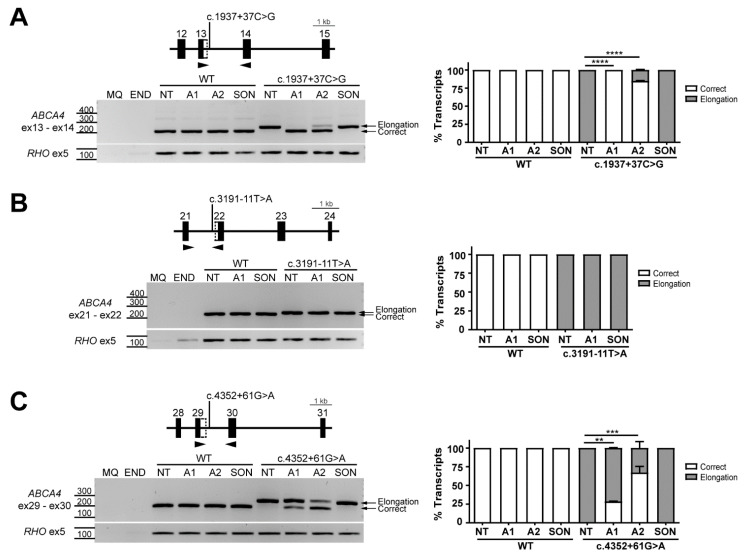
Antisense oligonucleotide (AON)-mediated rescue for near-exon splice variants causing exon elongation. Analysis of splicing correction by reverse transcription polymerase chain reaction (RT-PCR) upon AON (A#) delivery. Wild-type (WT) midigenes and the corresponding mutant midigenes containing near-exon variants c.1937+37C>G (**A**) c.3191-11T>A and (**B**) c.4352+61G>A (**C**) were transfected in HEK293T. Non-transfected HEK293T were used as endogenous expression control (END). The different AONs and a SON were then delivered, except for the non-treated lanes (NT). Genomic region of the different midigenes is depicted on top of each representative RT-PCR image, whereas the graphs next to each panel represent the semi-quantification of the resulting RT-PCR products, showing the percentage of correct and exon elongation transcripts. Exon elongation caused by two out of the three near-exon variants was efficiently rescued by at least one AON. MQ shows the negative control of the PCR reaction and amplification of exon 5 of the rhodopsin (*RHO*) gene was used as a transfection control. Data (n = 2) are presented as mean ± SD. Statistical significance is indicated as ** *p* < 0.01, *** *p* < 0.001 and **** *p* < 0.0001 using one-way ANOVA.

The three near-exon variants each generated 100% mis-spliced transcript without any detectable correct transcripts. The c.1937+37C>G variant is located in *ABCA4* intron 13 and creates a new strong cryptic splice donor site (SDS), causing a 36-nt exon elongation. This exon elongation introduces a premature stop codon (p.Phe647*) [13]. The c.3191-11T>A variant causes an exon extension including 9 nt upstream of the exon 22 SAS, leading to the incorporation of four new amino acids (p.(Gly1064delinsValProProGly)) [31]. The c.4352+61G>A variant strengthened the existing cryptic SDS, causing a 57-nt exon elongation and introduction of premature stop codon (p.Glu1452*) [17]. As mentioned before, two AONs were designed to modulate the effects of c.1937+37C>G and c.4352+61G>A, whereas only one AON was designed to reverse the effect of c.3191-11T>A (Table 1, Figure 1). All tested AONs did not cause mis-splicing events in the wild-type (WT) sequence for all three variants (Figure 2). The pathogenic exon elongation due to c.1937+37C>G was completely abolished with AON1 (100% correction) and almost completely abolished with AON2 (85% correction) (Figure 2A, Supplementary Figure S2A). The 9-nt exon elongation due to c.3191-11T>A was resistant to the effect of the AON as the correct transcript was not detected at all (Figure 2B, Supplementary Figure S2B). The failure to restore the correct transcript was likely the result of the limitations imposed by the region of interest sequence, which allowed the design of only a single AON. The last near-exon variant tested was c.4352+61G>A. AON1 and AON2 shared 14 nt of overlapping target sequence (Supplementary Figure S1). The AON2 outperformed AON1, however, neither were able to completely rescue aberrant splicing (AON1 showed 28% of correction whereas AON2 showed 67%) (Figure 2C, Supplementary Figure S2C). 

### 2.3. AON-Driven Splicing Modulation for Deep-Intronic Variants

**Figure 3:**
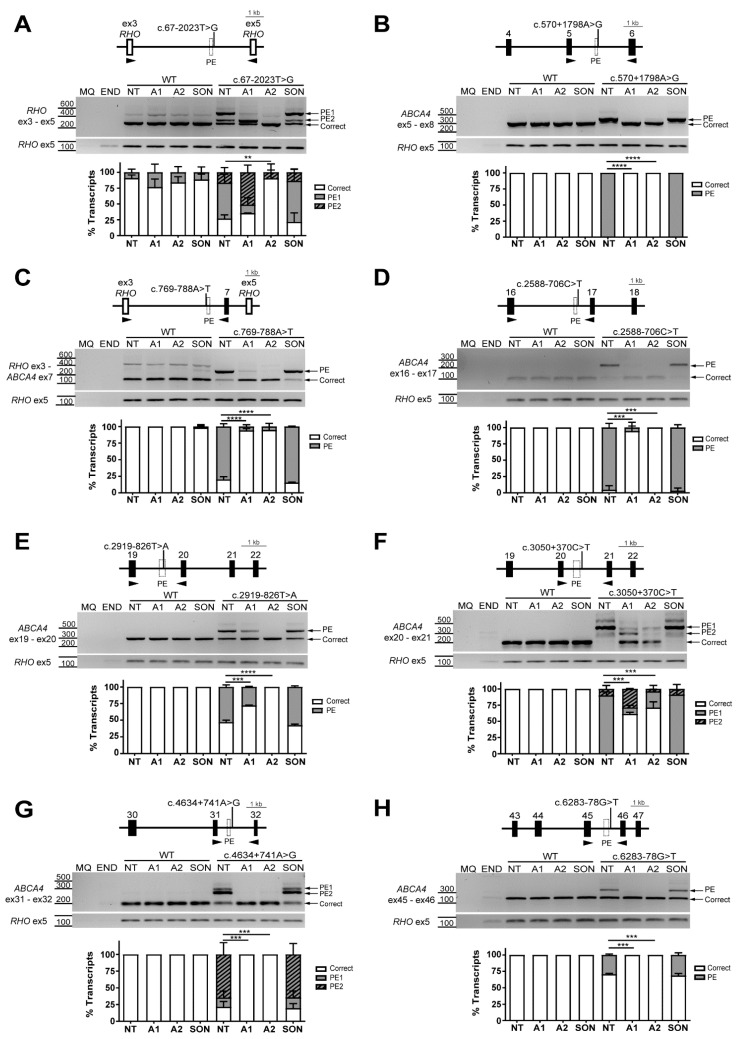
Antisense oligonucleotide (AON)-mediated rescue for deep-intronic splice variants causing non-overlapping pseudoexon (PE) inclusions. Analysis of splicing correction by reverse transcription polymerase chain reaction (RT-PCR) upon AON (A#) delivery. Wild-type (WT) midigenes and the corresponding mutant midigenes containing deep-intronic variants c.67-2023T>G (**A**), c.570+1798A>G (**B**), c.769-788A>T (**C**), c.2588-706C>T (**D**), c.2919-826T>A (**E**), c.3050+370C>T (**F**), c.4634+741A>G (**G**), and c.6283-78G>T (**H**) were transfected in HEK293T. Non-transfected HEK293T were used as endogenous expression control (END). The different AONs and a SON were then delivered, except for the non-treated lanes (NT). Genomic region of the different midigenes is depicted on top of each representative RT-PCR image, whereas the graphs below represent the semi-quantification of the resulting RT-PCR products, showing the percentage of correct and PE-including transcripts. For each deep-intronic variant causing PE inclusion, at least one AON could significantly restore 100% of the correct transcript, except for c.67-2023T>G and c.3050+370C>T. MQ shows the negative control of the PCR reaction and amplification of exon 5 of the rhodopsin (*RHO*) gene was used as a transfection control. Data (n = 2) are presented as mean ± SD. Statistical significance is indicated as ** *p* < 0.01, *** *p* < 0.001 and **** *p* < 0.0001 using one-way ANOVA.

For three variants, c.570+1798A>G, c.4634+741A>G, and c.6283-78G>T, creating a 65-nt, 127-nt, and 203-nt PE insertion, respectively [13] (Figure 1), all tested AONs were able to fully restore the correct transcript (Figure 3B,G,H; Supplementary Figure S3B,G,H). For each variant, two unique AONs targeted either the intron/PE boundary or high-scored SC35 sequence motifs located within the PE (Supplementary Figure S1). The c.2588-706C>T variant caused the insertion of a 134-nt PE in intron 16 [13] and the two AONs tested had a high rate of splicing correction (Figure 3D, Supplementary Figure S3D). Overall, AON1 almost completely eliminated the presence of the PE (94% of correction). For AON2, which targeted the strongest SC35 site within the PE, 100% of the correct transcript was achieved. The 162-nt PE introduced by the c.769-788A>T variant was almost completely excluded from the final transcript (AON1 and AON2 reached 92% and 93% of correction, respectively) (Figure 3C, Supplementary Figure S3C). 

The c.2919-826T>A variant is described to strengthen a cryptic SDS, causing the inclusion of a 133-nt PE into the final transcript [17]. Upon AON2 transfection, aberrant splicing was completely restored, showing a significant correction efficiency (100% correction) (Figure 3E, Supplementary Figure S3E). However, the observed splicing correction for AON1 was less efficient and only increased the correct transcript from 47% up to 72% (47% of correction) (Figure 3E). Interestingly, the difference in splicing correction seems to correlate with the targeted region. AON1 targets one SC35 and the newly created SDS by the c.2919-826T>A variant, whereas the target sequence of AON2 corresponds to a region containing a high-scored SC35 motif (Supplementary Figure S1). 

The c.67-2023T>G variant, located in intron 1 of *ABCA4*, is expected to cause an insertion of a 243-nt PE (from here on referred to as PE1) (Figure 1). Surprisingly, another intense lower band was also identified, which has not been described to date (Figure 3A). The other band represents a 46-nt long PE (from here on referred to as PE2). The PE2 uses the same cryptic SDS, which was created due to the c.67-2023T>G substitution. However, the PE2 SAS is located at c.67-2069, while the one of PE1 is at position c.67-2266 (Supplementary Figure S3A). The AON1 targeted the cryptic SAS for PE1 and the AON2 targeted the cryptic SAS for the PE2 (Supplementary Figure S1). AON1 and AON2 were able to diminish the presence of the PE1 down to 16% and 0% from 57% in the NT control, respectively. As expected, AON1 failed to correct the insertion of PE2. It was observed that reduction in PE1 due to AON1 resulted in an increase of PE2 intensity accompanied by a slight increase in the correct transcript, which was not statistically significant. AON2 completely eliminated the presence of the PE2 and increased the correct transcript from 27% in the NT control to 90% after the treatment. 

To correct aberrant splicing caused by the c.3050+370C>T variant, two different AONs were tested. The target sequences corresponded to two different SC35 motifs located in the predicted 205-nt PE inclusion, caused by the strengthening of a cryptic SDS (Supplementary Figure S1). However, a second PE inclusion was detected in the non-treated mutant condition right below the 205-nt PE inclusion (Figure 3F), which was not previously reported. This transcript showed a shorter PE of 111-nt, which uses a different SAS but shared the same SDS with the larger PE (Supplementary Figure S3F). Although the AON design for this region was based on the 205-nt PE inclusion, both AONs showed a significant rescue of the aberrant transcripts. More concretely, AON1 targets two SC35 motifs at the beginning of the 205-nt PE, resulting in a decrease of PE1 down to 10% and an increase of correct transcript up to 61%. In addition to that, the 111-nt PE transcript was increased up to 30% compared to mutant NT condition (Figure 3F). On the other hand, AON2 targets one SC35 motif in the middle of PE1 or at the beginning of PE2 (Supplementary Figure S1), which results in a 70% increase of the correct transcript and significant PE1 transcript decrease, not affecting the proportion of the PE2 (Figure 3F). 

**Figure 4:**
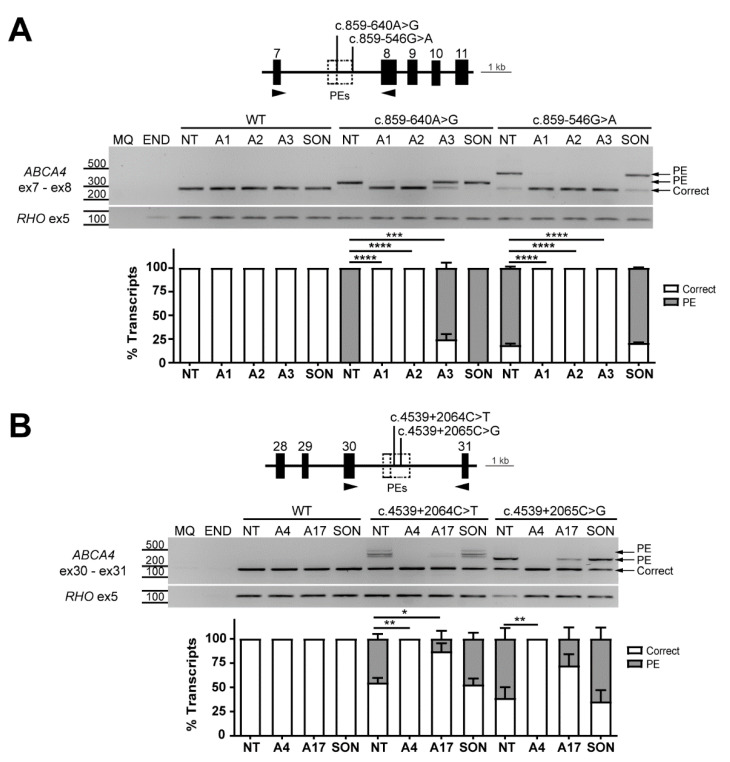
Antisense oligonucleotide (AON)-mediated rescue for deep-intronic splice variants causing similar pseudoexon (PE) inclusions in the same region. Analysis of splicing correction by reverse transcription polymerase chain reaction (RT-PCR) upon AON (A#) delivery. Wild-type (WT) midigenes and the corresponding mutant midigenes containing deep-intronic variants c.859-640A>G/c.859-546G>A (**A**) and c.4539+2064C>T/c.4539+2065C>G (**B**) were transfected in HEK293T. Non-transfected HEK293T were used as endogenous expression control (END). The different AONs and a SON were then delivered, except for the non-treated lanes (NT). Genomic region of the different midigenes is depicted on top of each representative RT-PCR image, whereas the graphs below represent the semi-quantification of the resulting RT-PCR products, showing the percentage of correct and PE-including transcripts. AON1 and 2 could rescue 100% of the correct transcript for both c.859-546G>A and c.859-640A>G variants, while AON4 completely redirected splicing in the case of c.4539+2064C>T and c.4539+2065C>G. MQ shows the negative control of the PCR reaction and amplification of exon 5 of rhodopsin (*RHO*) gene was used as a transfection control. Data (n = 2) are presented as mean ± SD. Statistical significance is indicated as * *p* < 0.05, ** *p* < 0.01, *** *p* < 0.001 and **** *p* < 0.0001 using one-way ANOVA.

The c.859-546G>A and c.859-640A>G variants are located in intron 7 causing insertions of overlapping PEs; 141 nt (c.859-546G>A) and 46 nt (c.859-640A>G) (Figure 1). The two variants use the same, strong intronic SAS located at c.859-685. However, the c.859-640G>A substitution created a new cryptic SDS. The c.859-546G>A substitution also created a new SDS at c.859-545. AON1 and AON2 were designed to correct the presence of both PEs. AON1 targeted the SAS used by both PEs and its 5′ end partially blocked an SC35 site. AON2 targeted the shared SC35 motif 17 nt downstream of the PE1 and PE2 SAS. AON3 was an AON designed only for c.859-546G>A as its complementary region is found outside of the PE due to c.859-640A>G (Supplementary Figure S1). The 141-nt PE was successfully skipped after treatment with all three AONs. For the 46-nt PE, AON1 and AON2 caused complete rescue of the aberrant transcript. AON3, whose complementary region is located outside of the 46-nt PE, had lower efficacy as it was expected (24% of correction) (Figure 4A, Supplementary Figure S4A).

Variants c.4539+2064C>T and c.4539+2065C>G are located in intron 30 next to each other, and share a proximal intronic region with the previously described c.4539+2001G>A (V4) and c.4539+2028C>T (V5) variants. We hypothesized that the most efficient AONs targeting the latter variants might also be capable of rescuing the 345-nt PE and the 170-nt PE caused by the variants c.4539+2064C>T (creating a new ESE) and c.4539+2065C>G (weakening an ESE and creating a new SDS). Therefore, AON4 and AON17 were chosen as the sequences with the highest correction potential [30,32]. As depicted in Figure 4B, AON17 showed a decrease of the 345-nt PE from 25% to 10%, and from 60% to 30% in the case of the 177-nt PE. On the contrary, AON4 was able to completely abolish the aberrant transcript generated by both deep-intronic variants. It is important to note that AON4 and AON17 target two different regions within the PE. Notably, AON17 overlaps with the region where the two variants c.4539+2064C>T and c.4539+2065C>G are located. This results in a one-nucleotide mismatch between the target region and the AON, which might explain the partial splicing correction that was observed (Figure 4A, Supplementary Figure S4A).

## 3. Discussion

During the last years, whole locus sequencing in STGD1 has revealed a remarkable amount of intronic variants in *ABCA4*. Recently, 15 new causative intronic variants have been reported. Using AON technology, we were able to correct the splicing defect introduced by 14 of these mutations.

More than a thousand disease-causing variants in *ABCA4* have been identified in STGD1 patients [7]. The size of the gene cDNA (approximately 6.88 kb) and this high allelic heterogeneity increase the difficulty to find a common treatment for the patient population and pose a challenge for therapeutic development. However, research on IRDs has opened many opportunities for the development of molecular therapies over the last several years. In fact, frequently reported intronic variants among STGD1 cases, which interfere with *ABCA4* splicing, have emerged as an attractive target for AON-based splicing-modulation therapy [37]. In this study, we designed and screened 26 different AONs targeting 15 recently described intronic variants that are fairly well distributed amongst different *ABCA4* intronic regions. The final aim was to rescue the aberrant splicing and select the most promising therapeutics to revert the STGD1 phenotype.

As reported in previous studies, AONs with the phosphorothioate (PS) backbone and 2′-OMe (2’-*O*-methyl) sugar modifications (2′-OMe-PS) can significantly correct aberrant transcripts in different IRD-associated genes [18,21,22,25,26,27,28,30,31,32]. To date, these modifications have given great resistance to nucleases and increase binding, and enhanced AON properties as drug-like molecules. Despite that, the 2′-*O*-MOE sugar modification has also been described as appropriate for therapeutic approaches, especially due to the increased resistance to nucleases and the reduced nonspecific protein binding which can reduce toxicities [38]. Moreover, a high splicing-switching capacity of the 2′-*O*-MOE-PS chemistry when delivered systemically has also been reported [39]. Until very recently, this modification was not commercially available, and it has rapidly been extendedly used for many antisense drugs that are now under clinical evaluation [40]. Therefore, due to recent evidence in antisense drug development, our AON design has been based on the implementation of 2′-*O*-MOE-PS chemistry for the in vitro screening. Last, variants c.4539+2064C>T and c.4539+2065C>G were targeted with the same antisense sequence but in 2′-*O*-MOE-PS instead of the previously used 2′-OMe-PS chemistry [30,32].

As shown in Figure 1, the design of AONs targeting near-exon variants is different and more challenging compared to the AONs targeting deep-intronic variants. For the near-exon variants, there is an important variant-dependency as the main objective is to block the newly created splice site, while not interfering with the binding of splice ancillary proteins to splicing enhancer motifs that facilitate original splicing. This step is crucial as blocking of regulatory regions may cause a failure in recognizing the exon, consequently leading to exon skipping and disrupting protein synthesis. In addition, the near-exon variants are more difficult to target as they are located in close proximity to the canonical splice sites of the natural exon. AONs in this region might block the canonical splice site and consequently inhibit the binding of the spliceosome and cause exon-skipping, which is not the type of splicing modulation aimed for *ABCA4*. In contrast, this exon/skipping strategy is used to rescue other alterations at pre-mRNA level. For instance, this approach has been of high utility in Duchenne muscular dystrophy, where AONs can efficiently restore the reading frame of the dystrophin gene by blocking splice sites and bypassing the target exons [41]. 

As an example, variant c.3191-11T>A was targeted using only one AON due to the above-mentioned limitations, and consequently, we could not rescue the exon elongation defect. The variant is in close proximity to *cis*-acting splice regulatory elements such as the SAS and the branchpoint and it is surrounded by a high density of splice enhancer motifs. The risk of blocking the aforementioned features with AONs could result in additional mis-splicing events such as exon skipping, and, therefore, only one AON was designed for this variant. However, future attempts to develop new AONs binding near the new SAS—instead of binding on top of the variant—might still help to avoid its recognition and to use the canonical SAS of exon 22. On the other hand, AONs targeting variants c.1937+37C>G and c.4352+61G>A could efficiently rescue exon elongation, showing how the distance relative to the canonical splice site significantly affects the correction efficiency. Nevertheless, the difference between blocking the new acceptor site and donor site should be noted due to recognition by the different spliceosome complexes at the 5′ or 3′ intronic regions [42], as the involved mechanism might have an impact on splicing modulation approaches. Recently, it has been shown that stereochemistry of the PS linkage can increase the potency of AONs [43,44]. In general, stereopure AONs can be more efficacious at a lower concentration. This could improve the efficacy in all AONs that successfully redirected splicing. Unfortunately, this concept might not be true for the c.3191-11T>A variant as the absent efficiency of the AON might be limited by the target region rather than the purity of the molecule.

Moving on to the deep-intronic variants, AON design was mainly focused on blocking the enhancer motifs within the PE region. Our results again confirm the high efficiency of this strategy to rescue aberrant PE-carrying transcripts, instead of blocking the newly created splice site. A clear example of this is variant c.2919-826T>A. Here, AON1 blocks the new SDS, whereas AON2 covers two high-scored SC35 motifs within the PE. Interestingly, only the second AON managed to fully restore correct transcript formation, whereas the AON1, which is a variant-specific AON, did not reach such correction. 

While PE inclusion was completely reverted in most of the cases by at least one of the designed AONs, 100% splicing correction could not be achieved for variant c.3050+370C>T. As already commented, the targeting of the 205-nt PE gave rise to the inclusion of a shorter PE, which was increased by the treatment with AON1 due to its binding to SC35 motifs only present in the larger PE. Considering that splicing is a dynamic and complex process [42,45,46,47], it is very difficult to predict alternative events that might be activated due to the use of splicing modulation molecules. In addition, splicing has been described as a non-sequential process by in silico real-time visualization approaches [48,49,50]. Therefore, the detection of new aberrant transcripts due to the use of AONs can make it more difficult to discern whether it is a real event or an event that is induced or enhanced by the in vitro delivery of AONs. Moreover, a shorter PE was also detected in the case of c.67-2023T>G and a larger one in the case of c.4634+741A>G, but both were completely corrected. Overall, these results indicate how important the position of enhancer motifs within the PE is in order to achieve high splicing correction. Nevertheless, these side mis-splicing effects could eventually be corrected by a novel AON design covering that region.

Notably, the ability of AON4 to effectively rescue mis-splicing caused by c.4539+2001G>A and c.4539+2028C>T in previous studies was translated to c.4539+2064C>T and c.4539+2065C>G variants as well [30,32]. This AON targets a common SC35 motif within the PE region associated with the four different variants, and because of their close proximity in the intron, these variants could be targeted with one single AON. Nevertheless, our experiments and earlier research differ in the chemistry of the oligo and the cell model. In the first case, 2′-OMe-PS chemistry was initially used for AON testing in patient-derived photoreceptor progenitor cells (PPCs) containing either the c.4539+2001G>A or the c.4539+2028C>T variant. The 345-nt PE inclusion was not observed in midigene-transfected HEK293T, and consequently, these variants were analyzed in patient-derived retina-like cells with endogenous *ABCA4* expression. In our case, c.4539+2064C>T and c.4539+2065C>G variants gave strong PE inclusion in HEK293T which was considered sufficient to be targeted with these AONs. However, it might also be interesting to confirm these observations by investigating these variants in a more retina-like model such as PPCs.

In addition to that, AON17 shows a mismatch with both c.4539+2064C>T and c.4539+2065C>G, whereas there are no mismatches when targeting the previously reported c.4539+2001G>A and c.4539+2028C>T variants (Supplementary Figure S5). The selection of AON17 was based on the described high efficiency in PPCs [32] and the position within both 345-nt and 170-nt PEs. The other potential candidates with no mismatches were discarded as they could only target the 345-nt PE introduced by c.4539+2064C>T, but not the 170-nt PE caused by c.4539+2065C>G. In contrast, AON4 was targeting a common region in both PEs. While AON4 fully corrected the splicing defect, AON17 only did it partially. When comparing the efficacy of these AONs based on the chemical modifications, we could indeed see that the 2′-*O*-MOE-PS AONs were more potent than the 2′-OMe-PS AONs (Supplementary Figure S5). This is in line with previous reports showing that the 2′-*O*-MOE-PS AONs have increased potency [39,51]. Previously, we reported that for this PE insertion, one mismatch was enough to abolish the splicing-switching capacity of the AON [32]. Nevertheless, this is not the case in this system. In the previous work, PPCs expressing *ABCA4* endogenously were used, while here we used an artificial overexpression system. Furthermore, AON17 fully binds to the PE carrying the mutation c.4539+2064C>T (Supplementary Figure S5). This could explain why AON17 performs better for this mutation, despite the mismatch. Taken all together, these two AON molecules are very promising to correct the splicing defects caused by several intronic variants.

Similar results have been obtained when testing AON1 and AON2 for overlapping PEs caused by c.859-546G>A and c.859-640A>G variants. This represents another clear example of a mutation-independent concept when designing AONs that can rescue the same splicing defect, caused by different mutations. Still, this strictly depends on the position of the PE and how many variants (if any) are detected in a certain intronic region. In conclusion, it is worth highlighting that the above-mentioned results were obtained with the midigene system and the efficiency of the AONs may be different from the efficiency in a retinal environment. Considering the simplified pre-mRNA folding in the midigene system, it is recommended to translate these assays to retina-like cellular models. 

The implications of this study in the current IRD treatment field are mainly related to the translational properties of AONs and their broad use in the clinical setting. Importantly, the allelic heterogeneity observed in STGD1 and the variant-dependent disease progression engages current research with world-wide *ABCA4* pathogenic variants identification and, in parallel, the development of therapies to halt or slow down vision loss. The clinical presentation of STGD1 is driven by a combination of variants, which can be grouped based on the proposed severity from deleterious to mild, many of them affecting splicing [13,32]. As a consequence, the combination of variants dictates the onset and progression of the disease [7].

Ongoing clinical trials are mainly focused on cell replacement therapy, compound administration and gene augmentation therapy [52,53,54,55,56,57]. Currently, AON-based therapies are extensively studied for *ABCA4*-associated retinopathies due to the high proportion of splice-affecting variants, yet none have entered clinical trials [18,30,31,32]. One of the most advanced clinical trials is the AON-based therapy targeting a recurrent deep-intronic mutation in *CEP290* [21,22,23,24,26], which showed improvement in vision during phase 1/2 and allowed the study to proceed to a phase 2/3 pivotal trial [58]. Hence, the increasing implementation of AONs in the clinical setting contributes to the development of new strategies for initial in vitro testing of the oligos, including more reliable cell models and simultaneously expanding the assessment of AON therapeutic efficacy beyond the RNA level.

As a result of the continuous discovery of pathogenic and rare intronic *ABCA4* variants, AON-based therapy can become part of the rising field of personalized medicine for IRD patients. Thus, this raises the opportunity to accelerate the treatment of rare diseases by normalizing N-of-1 clinical trials as previously carried out [59]. In conclusion, our results reinforce the use of splicing modulation approaches to effectively rescue the STGD1 phenotype in the future, making a significant contribution to the development of personalized medicine. 

## 4. Materials and Methods

### 4.1. AON Design

For each *ABCA4* intronic variant causing PE inclusion, two AONs were designed according to previous guidelines described elsewhere. Two particular variants, c.859-640A>G and c.859-546G>A, led to overlapping PEs and therefore three AONs were designed for this region of interest. For the exon elongation-causing variants, one or two AONs were designed aiming to block the new splice site [36,41]. For two variants, c.4539+2064C>T and c.4539+2065C>G, previously described AONs targeting the same region were used [30,32]. All sequences and properties of the different AONs are described in Table 1. The length, GC content and Tm of each AON were calculated using online software Oligo Calc: oligonucleotide properties calculator [60]. The oligonucleotides were purchased from Eurogentec (Liege, Belgium) and all of them had phosphorothioate (PS) backbone and 2’-*O*-methoxyethyl (2′-*O*-MOE) sugar modification. The PS modifications were not controlled for stereochemistry. The oligonucleotides were purified using reverse phase high-performance liquid chromatography to achieve purity of ≥85% according to the manufacturer. 

### 4.2. Generation of ABCA4 Mutant Midigenes

A new mutant construct was obtained for pathogenic variant c.3191-11T>A. To achieve that, site-directed mutagenesis was performed on wild-type BA16 [16]. Mutagenesis primers are listed in Supplementary Table S2. The mutagenesis PCR reaction mixture (50 μL) contained 0.5 μM of each primer, 0.2 mM dNTPs, 1 U Phusion High-Fidelity DNA Polymerase (New England Biolabs, Ipswich, MA, USA), 1× Phusion HF Buffer, 0.5x Q-solution (Qiagen, Venlo, The Netherlands), and 40 ng wild-type plasmid as template. Reaction conditions were set as follows: initial denaturation at 94 °C for 5 min; 15 cycles at 94 °C for 30 s each, annealing between 55 °C and 75 °C, and extension at 72 °C (1 min/kb), with a final extension at 72 °C for 20 min. PCR products were incubated with *Dpn*I (New England Biolabs) for 4.5 h at 37 °C, followed by heat inactivation at 65 °C for 20 min to digest the wild-type DNA. Subsequently, 5 μL of the digestion were transformed in DH5α. Upon picking colonies and plasmid isolation, the presence of the mutation was validated by Sanger sequencing (Supplementary Table S3). Lastly, the validated mutant constructs were cloned into the Gateway pCI-NEO-*RHO* destination vector as described in previous studies [16]. 

### 4.3. In Vitro Splice Assay in HEK293T Cells

Human Embryonic Kidney (HEK293T, ATCC# CRL-3216™) cells were cultured in DMEM supplemented with 10% FBS, 1% penicillin–streptomycin and 1% sodium pyruvate at 37 °C and 5% CO_2_. To check the effect on splicing of variant c.3191-11T>A in the newly generated mutant *ABCA4* midigenes, HEK293T were seeded in a 12-well plate and subsequently transfected with 600 ng of either the wild-type or mutant constructs when 70% confluent. Transfections were performed by using FuGENE HD reagent (Promega, Madison, WI, USA) following manufacturer’s protocol for the ratio 1:3 transfection. Forty-eight hours post-transfection, cells were harvested for transcriptional analysis by RT-PCR.

### 4.4. In Vitro AON Rescue Studies Using HEK293T Cells

To assess the efficacy of the AONs, HEK293T cells were seeded in 6-well plates at a confluence of 70%. After 4 h, when cells were attached, cells were transfected with 1 µg of the corresponding wild-type or mutant midigene, as well as a non-transfected well which acted as endogenous control. Midigene constructs and variant-harboring *ABCA4* regions are provided in Figure 2, Figure 3 and Figure 4. After overnight incubation, cells were trypsinized and divided into 5 wells of a 24-well plate. Oligonucleotides were resuspended at 100 µM in PBS. When completely attached (after 4 h), cells were transfected with 0.5 µM of the corresponding AONs, or the scramble-oligonucleotide (SON) as negative control. Another well was left non-treated (NT). Midigene transfections were performed as previously indicated. Delivery of the AONs was conducted by using 1 µL of FuGENE HD reagent and 0.5 µM of AON/SON in 50 µL of OptiMEM as previously described elsewhere [36]. Forty-eight hours post-AON delivery, cells were harvested for transcriptional analysis by RT-PCR. All experiments were performed in two independent replicates. 

### 4.5. RNA Isolation and RT-PCR Analysis

RNA was isolated from HEK293T cells using the Nucleospin RNA kit (Machery-Nagel, Düren, Germany) following the manufacturer’s instructions. One microgram of total RNA was used for cDNA synthesis using iScript cDNA Synthesis kit (Bio-Rad, Hercules, CA, USA) according to the provider’s protocol. All reverse transcription-PCR (RT-PCR) reaction mixtures (25 µL) contained 10 µM of each primer pair, 2 mM of dNTPs, 1.5 mM MgCl_2_, 0.5x Q-solution, 1 U of Taq polymerase and 60 ng of cDNA. PCR conditions were 94 °C for 30 s, followed by 35 cycles of 30 s at 94 °C, 30 s at 58 °C and 1 min at 72 °C, with a final extension step of 10 min at 72 °C. Exon 5 of *RHO* was used to assess the transfection efficiency of the midigene constructs. Primer sequences are listed in the Supplementary Table S4. PCR products were resolved on 2% agarose gels and products were validated by Sanger sequencing. Semi-quantitative analysis of the bands was performed by using Fiji software [61] (Supplementary Table S5). Percentage (%) of splicing correction was calculated from the decrease of aberrant transcript by the AON treatment relative to the corresponding non-treated mutant condition (Table 1).

### 4.6. Statistical Analysis

Data were represented as means ± SD and analyzed with GraphPad Prism 9 software (GraphPad, San Diego, CA, USA). To study the differences between treated and untreated conditions we implemented the one-way ANOVA test with subsequent Bonferroni correction. *p*-values smaller than 0.05 were considered statistically significant as indicated in the figures.

## 5. Patents

F.P.M.C., R.W.J.C. and A.G. declare that they are inventors on a filed patent (WO2018109011A1) that is related to the contents of this manuscript. The rest of the authors declare no conflicts of interest.

## Figures and Tables

**Table 1:** Antisense oligonucleotide (AON) sequences and characteristics for the 15 intronic *ABCA4* variants with the corresponding RNA defect.

Variant	AON#	Sequence (5′ to 3′)	L	Tm	GC	% Correction	RNA Defect
c.1937+37C>G	AON1	CCGUGUCAUGGAGGAGGAUC	20	55.9/64.0	60	100.00	36-nt exon elongation
	AON2	CCAUUACCGUGUCAUGGAGGA	21	54.4/49.2	52	84.64	
c.3191-11T>A	AON1	GCACUAGAAGGACGGGAG	18	52.6/58.0	61	0.00	9-nt exon elongation
c.4352+61G>A	AON1	GAACUCACCGUUGGGUCCU	19	53.2/60.0	58	28.40	57-nt exon elongation
	AON2	UCUUGAACUCACCGUUGG	18	48/54.0	50	67.07	
c.67-2023T>G	AON1	UGCGGCAACAUCUAUCUGG	19	51.1/58.8	53	11.47	243-nt PE inclusion
	AON2	CAUCAGUGGGUAAGGCUG	18	50.3/56.0	56	87.54	
c.570+1798A>G	AON1	CUGGAAGUCAUCAAGGCAUUG	21	52.4/47.3	48	100.00	65-nt PE inclusion
	AON2	GACUUGAGUUUUACGAGCUG	20	49.7/58.0	45	100.00	
c.769-788A>T	AON1	GGAAUCACUGAUCCUAGAGG	20	51.8/60.0	50	92.72	162-nt PE inclusion
	AON2	GGAUGUGGAAGUCCCCAGG	19	55.4/62.0	63	93.11	
c.859-640A>G	AON1	CCAGUUCUUGGGUUCUGUUG	20	51.8/60.0	50	100.00	46-nt PE inclusion
	AON2	CACCAAGAUGGGGAUACUGG	20	53.8/62.0	55	100.00	
	AON3	CCUCUCUUCUUCUAGUCUCC	20	51.8/60.0	50	24.34	
c.859-546G>A	AON1	CCAGUUCUUGGGUUCUGUUG	20	51.8/60.0	50	100.00	141-nt PE inclusion
	AON2	CACCAAGAUGGGGAUACUGG	20	53.8/62.0	55	100.00	
	AON3	CCUCUCUUCUUCUAGUCUCC	20	51.8/60.0	50	100.00	
c.2588-706C>T	AON1	ACUGGACUGUCUAUUCCUCG	20	51.8/60.0	50	93.83	134-nt PE inclusion
	AON2	UCUUUAUCUCCACCGCUCUG	20	51.8/60.0	50	100.00	
c.2919-826T>A	AON1	CAGCUCUCUGACCUUAUCAGU	21	52.4/47.3	48	47.33	133-nt PE inclusion
	AON2	GCUCUGUCCCUGAGUUCUG	19	53.8/60.0	58	100.00	
c.3050+370C>T	AON1	CAGAGUCCCAUAUUCUCAGG	20	51.8/60.0	50	61.28	205-nt PE inclusion
	AON2	GGAUCGAUCAGCUGCUCUG	19	53.2/60.0	58	71.07	
c.4539+2064C>T	AON4	GGGGCACAGAGGACUGAGA	19	55.4/62.0	63	100.00	345-nt PE inclusion
	AON17	GCCAAGAGCUCAGGGUACAG	20	60/64.0	55.9	72.35	
c.4539+2065C>G	AON4	GGGGCACAGAGGACUGAGA	19	55.4/62.0	63	100.00	170-nt PE inclusion
	AON17	GCCAAGAGCUCAGGGUACAG	20	60/64.0	55.9	55.77	
c.4634+741A>G	AON1	UCUGAUACGGGCUGCCAAAG	20	53.8/62.0	55	100.00	127-nt PE inclusion
	AON2	UCCUUAGGAUCCUCUCUCCU	20	51.8/60.0	50	100.00	
c.6283-78G>T	AON1	GGCAAUGACAGAAUUCUCCUC	21	52.4/47.3	48	100.00	203-nt PE inclusion
	AON2	GCUGACAGAAGGCGCACAC	19	55.4/62.0	63	100.00	
N/A	SON	CGCCAAUUGCAAGGUGAUUCC	21	54.4/49.2	52	N/A	N/A

L: Length in nt; Tm: theoretical melting temperature in °C calculated with OligoCalc (left) and provided by the manufacturer (right); GC: percentage of GC; % Correction: percentage of observed splicing correction for each AON.

## Data Availability

The data supporting all our results are provided in this manuscript either as a main figure or Supplementary Materials.

## Supplementary Materials

The following are available online at https://www.mdpi.com/article/10.3390/ijms22094621/s1, Figure S1: Genomic regions of interest used for antisense oligonucleotide (AON) design, Figure S2: Sequence analysis of the resulting RT-PCR products from rescue experiments of near-exon variants causing exon elongation, Figure S3: Sequence analysis of the resulting RT-PCR products from rescue experiments in deep-intronic variants causing not overlapping pseudoexon (PE) inclusion, Figure S4: Sequence analysis of the resulting RT-PCR products from rescue experiments in deep-intronic variants causing similar pseudoexon (PE) inclusion in the same region, Figure S5: Aberrant splicing rescue for c.4539+2064C>T and c.4539+2065C>G deep-intronic variants by 2’-OMe-PS AON4 and AON17. (A) Analysis of splicing correction by reverse transcription polymerase chain reaction (RT-PCR) upon AON delivery. Wild-type (WT) midigenes and the corresponding mutant midigenes containing variants c.4539+2064C>T and c.4539+2065C>G were transfected in HEK293T, leaving one empty as endogenous HEK293T expression control (END). The different AONs and a SON (2’-OMe-PS) were then delivered, except for the non-treated lanes (NT). Genomic region of the different midigenes is depicted on top of each representative RT-PCR image, whereas the graphs next to each panel represent the semi-quantification of the resulting RT-PCR products, showing the % of correct and pseudoexon (PE) inclusion transcripts. MQ shows the negative control of the PCR reaction and amplification of exon 5 of Rhodopsin (RHO) gene was used as a transfection control. Data (n = 2) are presented as mean ± SD (* *p* < 0.05, ** *p* < 0.01, *** *p* < 0.001, **** *p* < 0.0001). (B) Predicted binding of AON17 on target pre-mRNA presenting c.4539+2064C>T or c.4539+2065C>G variants (predictions were obtained using RNA structure software: http://rna.urmc.rochester.edu/RNAstructureWeb/, accessed on 20 April 2021). Table S1: Primer sequences for site-directed mutagenesis, Table S2: Primer sequences for sequencing entry clones, Table S3: Primers for RT-PCR analysis, Table S4: Semi-quantification analysis of transcripts from RT-PCR products. Table S5: Semi-quantification analysis of the RT-PCR products from non-treated (NT) or AON/SON-treated samples. The amount of the different transcripts is represented as a percent (%) of the total transcript for each condition or lane. The average of two independent experiments, followed by two independent semi-quantification for each replicate is provided. SD: standard deviation.
