# Supplementary material for: Antisense Oligonucleotide-Based Rescue of Aberrant Splicing Defects Caused by 15 Pathogenic Variants in ABCA4
Source: Int J Mol Sci. 2021 Apr 28;22(9):4621. doi: 10.3390/ijms22094621 (PMC8124656; doi:10.3390/ijms22094621)
Supplement: Supplementary file 1 [file ijms-22-04621-s001.zip › ijms-1187288-supplementary.pdf]

Supplementary Figure S1. Genomic regions of interest used for antisense oligonucleotide (AON) design. Position of the antisense sequences (underlined), exon elongations or pseudoexon (PE) inclusions (grey), enhancer splicing motifs (green) and variants (yellow) are indicated for every analyzed region.

c.1937+37C>G

CAGGGGAUCACAAGGAGCCAGGUGCAGGCGGAGGCUCCAGUUGGAAUCUACCUCCAGCAGAU
GCCCUACCCCUGCUUCGUGGACGAUUCGUGAGUCUGAAGUUCGCGAUCCUCCUCCAUGACAC
GGUAAUGGGGGUGCUGGAGUGGGCUGGGGUGGGCUGGGGGUGCCCUCAAGGCUUCCAUGU
CUUUAGAGAGAGCCCCAGGGACCAGAGCCAAAUUGGAGAGCAUGGAGCUCUGACUGAGGAAC
CUGC

Exon 13

Exon elongation: r.1937_1938ins1937+1_1937+36 (36-nt elongation at SDS)

AON1: 5'- CCGUGUCAUGGAGGAGGAUC-3' (target sequence: GAUCCUCCUCCAUGACACGG)

AON2: 5'- CCAUUACCGUGUCAUGGAGGA-3' (target sequence: UCCUCCAUGACACGGUAAUGG)

c.3191-11T>A

ACGCUAUAAACAUCUAAGAGGCAGCACCAAACCACUGCUGGGUUAAGGUACCCCCACAAUGCC
ACUUGCCCUGGGCCUUUCUCUUCCUCACCCUCCACAGCCCCUUAACUCUCCCGUCCUUCUAG
UGCCUCCAGGUGGCAUGCAGAGAAAGCUGUCGGUUGCCAUUGCCUUUGUGGGAGAUGCCAAG
GUGGUGAUUCUGGACGAACCCACCUCUGGGGUGGACCCUUACUCGAGACGCUCAAUCUGGGA
U

Exon 21

Exon elongation: r.3190_3191ins3191-1_3191-9 (9-nt elongation at SAS)

AON1: 5'-GCACUAGAAGGACGGGAG-3' (target sequence: CUCCCGUCCUUCUAGUGC)

c.4352+61G>A

CAGACGUCCUCCUGAAUAAGCCAGGCUUUGGCAACCGCUGCCUGAAGGAAGGGUGGCUUCCG
UAAGUGCCUACGCGCCCCUGUCCUAAGAAGACUAGCUCCCCUGGGAGGACCCAACGGUGAGU
UCAAGAUGGCAGGCGUUGGGGAGGCCCCACUCAAUCCUGCUCUGCUGGUCACUUCCAUGUCU
CUGACCAGCACUCCCCCAACCUCUCCUUCCACACUUGUGUGCAGGGACAUUCACUACCUCCUA
G

Exon 29

Exon elongation: r.4352_4353ins4352+1_4352+57 (57-nt elongation at SDS)

AON1: 5'-GAACUCACCGUUGGGUCCU-3' (target sequence: AGGACCCAACGGUGAGUUC)

AON2: 5'-UCUUGAACUCACCGUUGG-3' (target sequence: CCAACGGUGAGUUCAAGA)

c.67-2023T>G

AGACUCAGAGGUUAAGCAAGCCCCAAAGUGGUUGUUAACCCAGAUCUUCCCACUAACUCCCAA
AUCAGCAUCAGUGUUUAACGUACCAGACCUCUCCCAGAUAGAUGUUGCCGCAUGGAAGACAGC
CGAUCUACGUGAUAGAAAGCCAAUAUUGCAAGCAGUCGUCUAAAGGAGUCAAAUGUGUUGGAU
UUGAACUGGAUGUCUCAUUUCUUUGGUGAAGACACUGGAAACAACUUCCAGGUUUCAUCAAUU
GCUCCUAUCACUCAACGUUGCUAUCUUACUGAACUUGUUCCCCAGCCUUACCCACUGAUGGAA
UGAUCCAGAAUGGAAGACAAGACACCAAGGUACAUGACCCUGGGGGAGGCUGUUUCUUAAAUC
UACAGACUGUUGGUGACCUGAGCCCCAUGUCACCAAAGGCUUUCCUGGAGAAGCCUCCUAGA
CCAGUCUUGACAAAGGCUCACUCAUUCCG

SC35 motif

PE inclusion: r.66_67ins66-2266_66-2023 (PE 243 bp)

AON1: 5’-UGCGGCAACAUCUAUCUGG-3’ (target sequence: CCAGAUAGAUGUUGCCGCA)

AON2: 5’-CAUCAGUGGGUAAGGCUG-3’ (target sequence: CAGCCUUACCCACUGAUG)

c.570+1798A>G

CCCCACAGAGGGGGUGCUGUCGGCACCGAUUGCUCAUUUUCCCCUUUGCUUUCUCUUCAGCU
CGUAAAACUCAAGUCCUGACAAUGCCUUGAUGACUUCCAGUUGGUAAUAAAAGGGAGAUGAAG
GUAAGGACAGGAAUUUCGGGGAAAUUUCUUUCCAGUUCCUUACUAAUGUGACAUUUAGAUCUC
UAGUACUGUGCUUCUGGCAUCAGUGCCAAGGCCUUUCAUGUUGGAGAAUGGAGGCCGGGGUC

SC35 motif

PE inclusion: r.570_571ins570+1733_570+1797 (PE 65 bp)

AON1: 5’-CUGGAAGUCAUCAAGGCAUUG-3’ (target sequence: CAAUGCCUUGAUGACUUCCAG)

AON2: 5’-GACUUGAGUUUUACGAGCUG-3’ (target sequence: CAGCUCGUAAAACUCAAGUC)

c.769-788A>T

UUGGAUGAGAAUUACAAGUCCAGCUGGUUAAAAGGCACAUGCCCAGUGCUCACUUCACACCUA
CUCAGGAAGCACACUUGAGUUGGAAAACCACUGUCUUUACACUUAGAACUCAGUCCUACAUGU
CUCCUCUAGGAUCAGUGAUUCCAUCAGUUUUGAAACAUGAAGCAUGAAGUCAAACAGGACAUG
ACCUUGGUUUCCAGAAAACCAGAUGUUCACAUCAGUCUCUGGAGCUUGGAGGCAGCACACCU
GGGGACUUCCACAUCCCCUGCCGAGGUGGCAAAAGCAGGAGCAGUGGUGAGUUCACAUGGGC
UGGGGUUUCCUGAACACUGCUGGCAAUUGGAGAAUCUGCAAGGGAACUUCUCCGACUCCUAC
CAGCAGCUGCUUUAAAAUAAAGGUGAUGUAGCUGGUCAAAUCCUCCA

SC35 motif

PE inclusion: r.768_769ins769-778_769-617 (PE 162 bp)

AON1: 5’-GGAAUCACUGAUCCUAGAGG-3’ (target sequence: CCUCUAGGAUCAGUGAUUCC)

AON2: 5’-GGAUGUGGAAGUCCCCAGG-3’ (target sequence: CCUGGGGACUUCCACAUCC)

c.859-640A>G

AACUAAAUUUUAGACUAUUUAGCCAAAGAAAUUUGCUCUGAUCUUGCUUUUCUACAACAGAAU
CAUUCCCCAAUCAUUUUAUUUCCCUCUUUUUCUCCCCAGUAUCCCCAUCUUGGUGGGACAACA
GAACCCAAGAACUGGCUUAACGGUAAAAUAUUUCUGCAUUUGCCCAAGGACACAUUCCCAAC
GAAUUCAAAUAAAGGAGACUAGAAGAAGAGAGGCUAUACUACAGUGCUCUAGGGGUCACUC

c.859-546G>A

AAUAUAAGAAACUAAAUUUUAGACUAUUUAGCCAAAGAAAUUUGCUCUGAUCUUGCUUUUCUAC
AACAGAAUCAUUCCCCAAUCAUUUUAUUUCCCUCUUUUUCUCCCCAGUAUCCCCAUCUUGGUG
GGACAACAGAACCCAAGAACUGGCUUAACAGUAAAAUAUUUUCUGCAUUUGCCCAAGGACACA
UUCCCAACGAAUUCAAAUAAAGGAGACUAGAAGAAGAGAGGCUAUACUACAGUGCUCUAGAGG
UCACUCUGUGAUUUGUUGUUGUUGUUGUUGUUGUUUUGAGACGGAGUAUUGCUCAGUCGCCC
AGGCUGGAGUGCAGUGGCACGAUGUCUACUCACUGUAAGCUCUGCCCCCCAGGUUCACGCC

SC35 motif

PE inclusion: r.858_859ins859-685_859-640 (PE 46 bp)

PE inclusion: r.858_859ins859-545_859-685 (PE 141 bp)

AON1: 5’-CCAGUUCUUGGGUUCUGUUG-3’ (target sequence: CAACAGAACCCAAGAACUGG)

AON2: 5’-CACCAAGAUGGGGAUACUGG-3’ (target sequence: CCAGUAUCCCCAUCUUGGUG)

AON3: 5’-CCUCUCUUCUUCUAGUCUCC-3’ (target sequence: GGAGACUAGAAGAAGAGAGG)

c.2588-706C>T

CUAUCCUACUAGAUUGUUGGAAACAGAUAUAAUAGAUGAAAAUUAGUUGAUUAAAAUUGAAAUU
UGUGCAUAAUUCAAAAGUUUUAUUUUAGCCAAGCUAAAGCUUUCAUUUAUUCAACAGCUAUUU
ACUGAGCAGCACCUGUGCAUGAGGCUCAGCAGGGCCAGGUUCUGGGAACAGAGCGGUGGAGA
UAAAGAUCCAGACCUGCCCCGAGGAAUAGACAGUCCAGUGGCAGCAAAGGCCAUGAAACAUAC
GGUAACUCUUAAAAAAAGCCGAGACCAUGAUUUUACAAAAUCAACAUUUUGUAGGGAGCAGAA
CUUUC

SC35 motif

PE inclusion: r.2587_2588ins2588-839_2588-708 (PE 134 bp)

AON1: 5’-ACUGGACUGUCUAUUCCUCG-3’ (target sequence: CGAGGAAUAGACAGUCCAGU)

AON2: 5’-UCUUUAUCUCCACCGCUCUG-3’ (target sequence: CAGAGCGGUGGAGAUAAAGA)

c.2919-826T>A

AUGUGGGGCCGGUGCCAUUCUAAUUGGUUCUGUUUCCCUCUAGUGGUUGAUCGCGGAGAUU
UCGGCUUCUCCAUCAGGACAAGUUCAGAUAGCCUGAGAUGGUAUCAGAACUCAGGGACAGAG
CUGGGUGUGGCGGCCCUGCAUCCAUCUGCUUUCUCUCCAUGCUAACUGAUAAGGUCAGAGAG
CUGGAAGCAAAUUCCAGGACCCCAGGGCUCCGCAAAGGCAAACACAUUACUUCAUCGGCUGCU
GA

SC35 motif

PE inclusion: r.2918_2919ins2919-957_2919-825 (PE 133 bp)

AON1: 5’-CAGCUCUCUGACCUUAUCAGU-3’ (target sequence: ACUGAUAAGGUCAGAGAGCUG)

AON2: 5’-GCUCUGUCCCUGAGUUCUG-3’ (target sequence: CAGAACUCAGGGACAGAGC)

c.3050+370C>T

GUCCUUUGUCCUACUUGCAGUGAGAAUUGUCCCUGAGAAUAUGGGACUCUGCCUCUGCUGCU
CAGAGCUGAGGGCUCCUCCCUCAGAAGGGUGAGGCUGCCUUCGCUCUGACAGAGCAGCUGAU
CGAUCCCCGAGCCCCUUGUGCAGCCCUGAAGUACUUCCUCUCUGGGACCAAAGACAGGAGAA
CCAUUGUUCCUUUUUCCUGUUGAAGCCACGGCCUGAAAGGUAAACUUUUCAGGGGGCUUUUC
AG

SC35 motif

PE inclusion: r.3050_3051ins3050+164_3050+368 (PE 205 bp)

AON1: 5’-CAGAGUCCCAUAUUCUCAGG-3’ (target sequence: CCUGAGAAUAUGGGACUCUG)

AON2: 5’-GGAUCGAUCAGCUGCUCUG-3’ (target sequence: CAGAGCAGCUGAUCGAUCC)

c.4539+2064C>T

UUAUAUUUCUCAAGCAAUAUUUUCUCUCUUUGAAUCACAGCUCAUCUGCUGCAUCAUAGGGAU
CCCAAAAGAAGGACCCAAGGAACUUGUCUCAGUCCUCUGUGCCCCAAGAGGAAGCUUUGCUU
GUUUGCUUUGCUGUCAAUGCUGAGG**G**CUCCUGUGGCUGCCUCCACUCAAAAC**C**CUCCAGCAU
CAGGACGUCAAGGCUGUGAUACUGUAUCCUGAGCUCUUGGCCAGGGCGAGGGAGGGGAGGC
CAAGCCUACCUACAUGGUGUUUCAUUUCCUAAACGAACCCUUACUUCCACGCGGUCUGUCCAG
CUUAGAAACUUAUUUUCAGUAGUGUUGGUCCUUGGUCCCUGGACAAAAUGUAACAGCCAAAGU
CCUAGAAAAAGGCAAGCCAGUUCCUGCCAUUUUCUUUCACUUCUGCAUUUCC

c.4539+2065C>G

UUAUAUUUCUCAAGCAAUAUUUUCUCUCUUUGAAUCACAGCUCAUCUGCUGCAUCAUAGGGAU
CCCAAAAGAAGGACCCAAGGAACUUGUCUCAGUCCUCUGUGCCCCAAGAGGAAGCUUUGCUU
GUUUGCUUUGCUGUCAAUGCUGAGG**G**CUCCUGUGGCUGCCUCCACUCAAAAC**C**CUCCAGCAU
CAGGACGUCAAGGCUGUGAUACUGUACGCUGAGCUCUUGGCCAGGGCGAGGGAGGGGAGGC
CAAGCCUACCUACAUGGUGUUUCAUUUCCUAAACGAACCCUUACUUCCACGCGGUCUGUCCAG
CUUAGAAACUUAUUUUCAGUAGUGUUGGUCCUUGGUCCCUGGACAAAAUGUAACAGCCAAAGU
CCUAGAAAAAGGCAAGCCAGUUCCUGCCAUUUUCUUUCACUUCUGCAUUUCC

SC35 motif

PE inclusion: r.4539_4540ins4539+1891_4540-2162 (345 bp)

PE inclusion: r.4539_4540ins4539+1891_4540-2060 (170 bp)

AON4: 5’-GGGGCACAGAGGACUGAGA-3’ (target sequence: UCUCAGUCCUCUGUGCCCC)

AON17: 5’-GCCAAGAGCUCAGGGUACAG-3’ (target sequence: CUGUACCCUGAGCUCUUGGC)

c.4634+741A>G

CAGGUCAACUGCAGCUGUCAUAUUCUAACUGUGAAUUAUCAUCUUUGAUCACUGCCCUUUGAG
AUGCCAAUGAACUUUUCAAGAAAUAUCUAGUUCUCUUGGCUCUCCAGCUGUUCUUAUCAGCCC
CAUCCAGGAUGGAACAGCUUUGGCAGCCCGUAUCAGAACAAGCAGCUUGACAGGGGCAUGCC
AUGCCAGGAGAGAGGAUCCUAAGGAAGCGUGGUCCAGUCCGCACAGGCUCUGGGGCUUUAAG
GUAAAACCUCCUGUCUAACUUUAGUAGGACUUUCUGUUGCUUCACCUGCCAGAGCCCUGAAC
GAGGGAUAAAUUGACUUAAUUAACUAGAACACACUGCAAAUGGUGAAAGCAUUUAGCAAAA

SC35 motif

PE inclusion: r.4634_46354ins634+614_4634+740 (PE 127 bp)

AON1: 5’-UCUGAUACGGGCUGCCAAAG-3’ (target sequence: CUUUGGCAGCCCGUAUCAGA)

AON2: 5’-UCCUUAGGAUCCUCUCUCCU-3’ (target sequence: AGGAGAGAGGAUCCUAAGGA)

c.6283-78G>T

GCCUUCUGACAAAUGUAUUUGCAGGAUUUUCUUUUUUUGAGGAGAAUUCUGUCAUUGCCUUA
AUCCACUUUAAUCCCCUCGUGGGCUGAAAUGGGCCCAGGAUGGACGCCACGCUUCUUUACUC
UUGGAUCCACCUCCUGCCUUCCCUACCCUACACCAGGGUACCCCUGUCUUGCUCAAGUGAGG
GGAGUGACUGUGUGCGCCUUCUGUCAGCUCAUCCUCCACAGGUGAGCCAGCCCAGGGGGAAG
CA

SC35 motif

PE inclusion: r.6283_6283ins6283-282_6283-80 (PE 203 bp)

AON1: 5’-GGCAAUGACAGAAUUCUCCUC-3’ (target sequence: GAGGAGAAUUCUGUCAUUGCC)

AON2: 5’-GCUGACAGAAGGCGCACAC-3’ (target sequence: GUGUGCGCCUUCUGUCAGC)

Supplementary Figure S2. Sequence analysis of the resulting RT-PCR products from rescue experiments of near-exon variants causing exon elongation. Sanger sequencing chromatograms from splicing correction experiments targeting c.1937+37C>G (**A**), c.3191-11T>A (**B**) and c.4352+61G>A (**C**) variants. For each case, sequences of the correct transcript from wild-type *ABCA4* midigene condition (**i**), AON-corrected transcript in mutant *ABCA4* midigene condition (**ii**) and exon elongation transcript in mutant *ABCA4* midigene condition (**iii**) are shown.

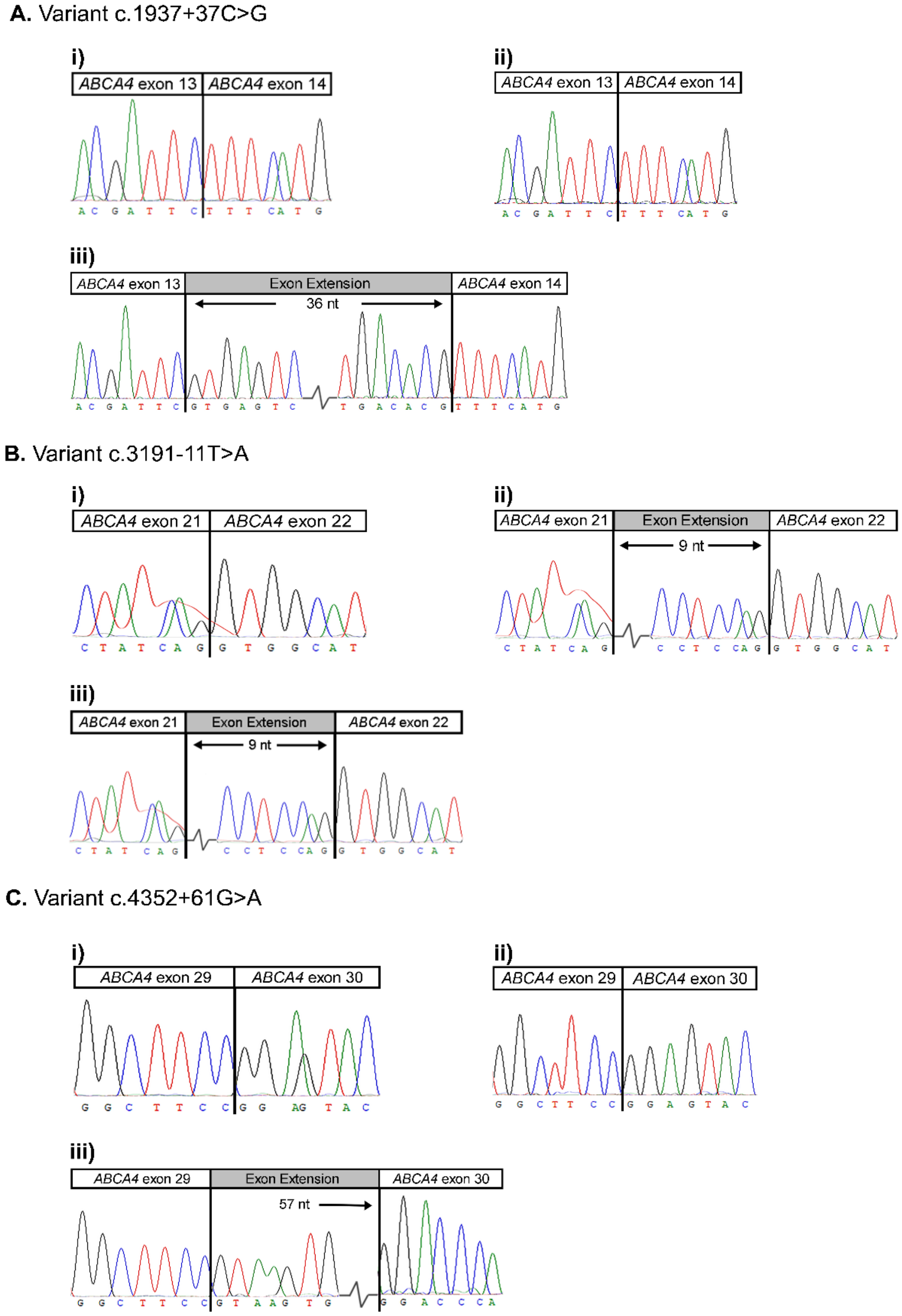

Supplementary Figure S3. Sequence analysis of the resulting RT-PCR products from rescue experiments of deep-intronic variants causing not overlapping pseudoexon (PE) inclusion. Sanger sequencing chromatograms from splicing correction experiments targeting c.67-2023T>G (**A**), c.570+1798A>G (**B**), c.769-788A>T (**C**), c.2588-706C>T (**D**), c.2919-826T>A (**E**), c.3050+370C>T (**F**), c.4634+741A>G (**G**) and c.6283-78G>T (**H**) variants. For each case, sequences of the correct transcript from wild-type *ABCA4* midigene condition (**i**), AON-corrected transcript in mutant *ABCA4* midigene condition (**ii**) and PE inclusion transcript in mutant *ABCA4* midigene condition (**iii-v**) are shown.

A. Variant c.67-2023T>G

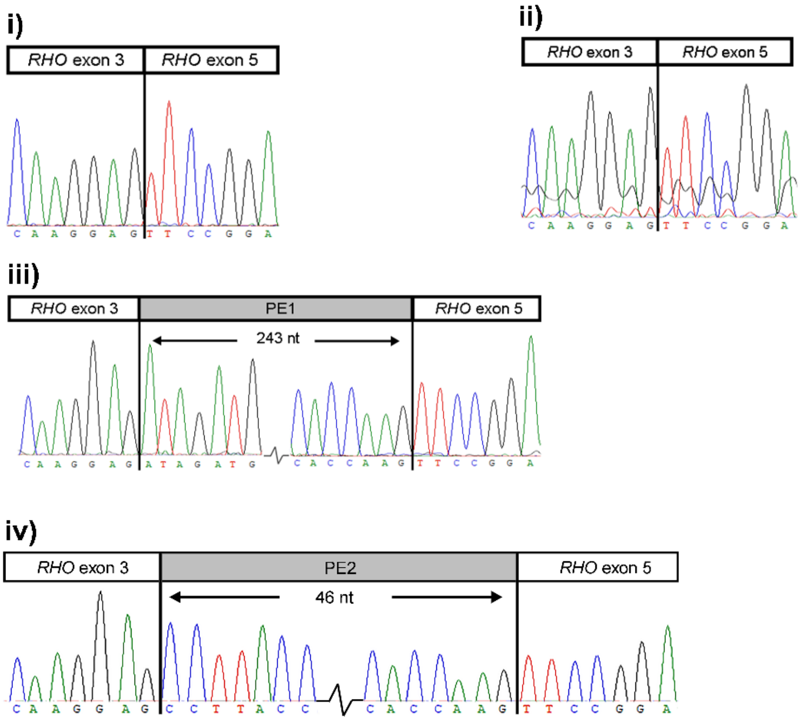

B. Variant c.570+1798A>G

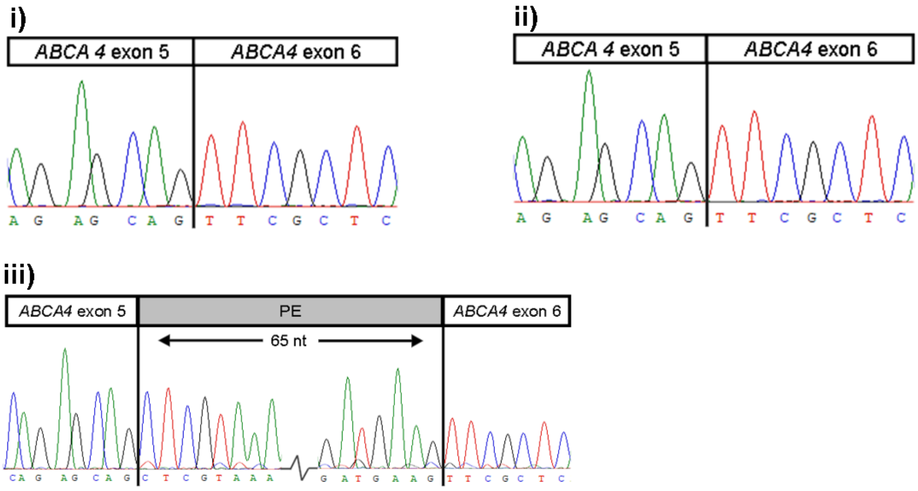

C. Variant c.769-788A>T

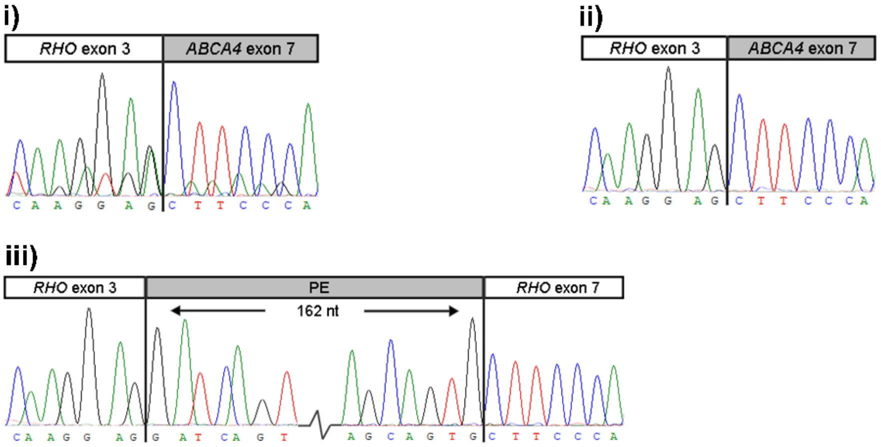

D. Variant c.2588-706C>T

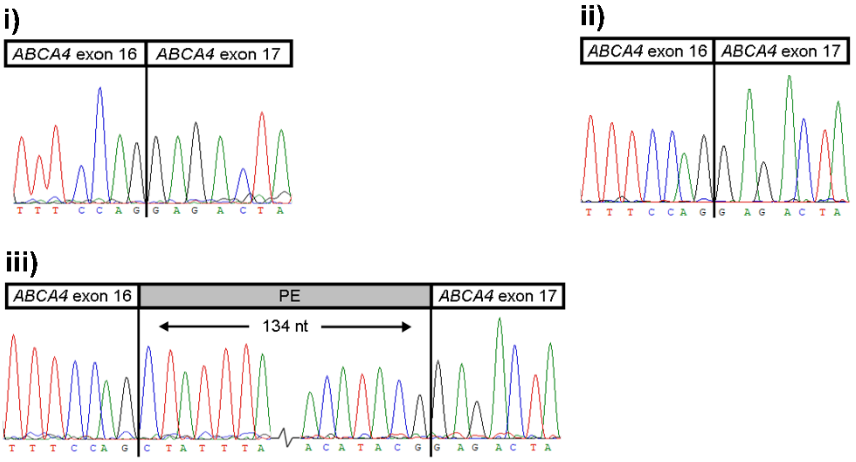

E. Variant c.2919-826T>A

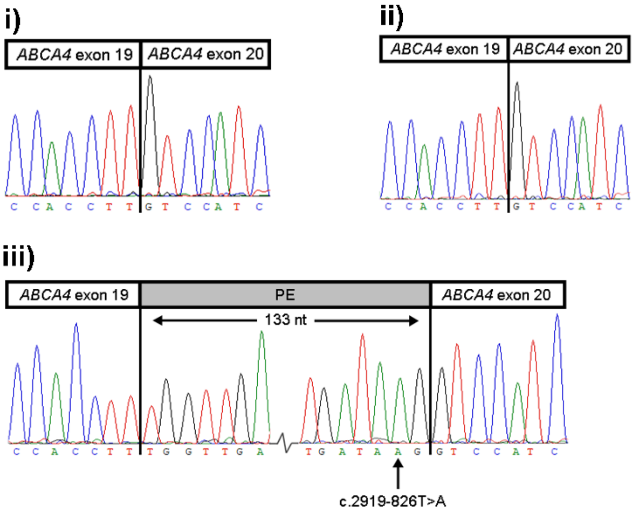

F. Variant c.350+370C>T

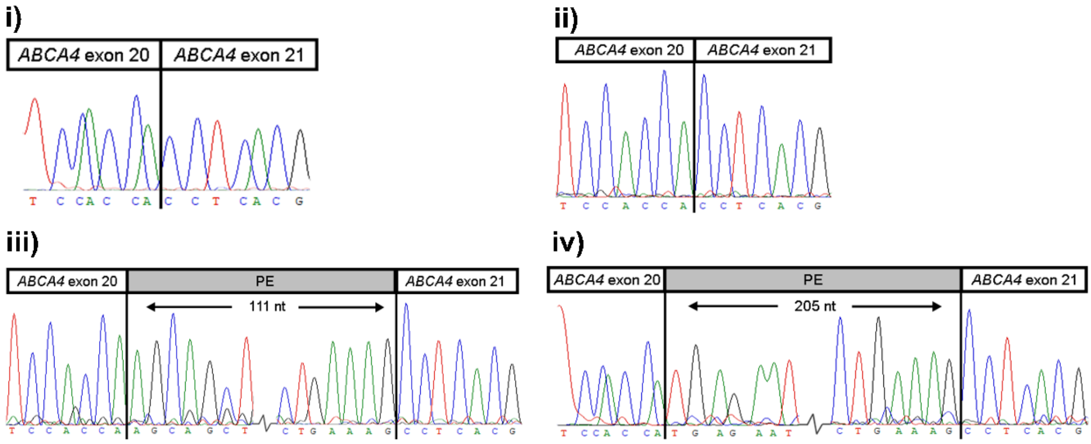

G. Variant c.4634+741A>G

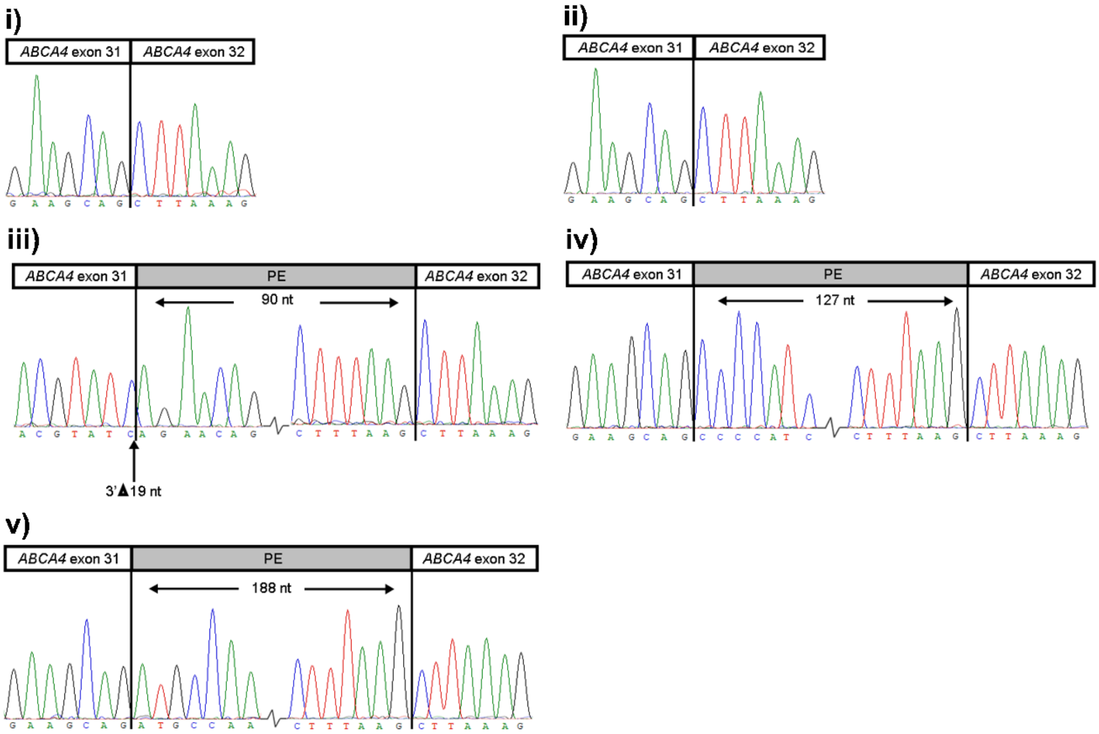

H. Variant c.6283-78G>T

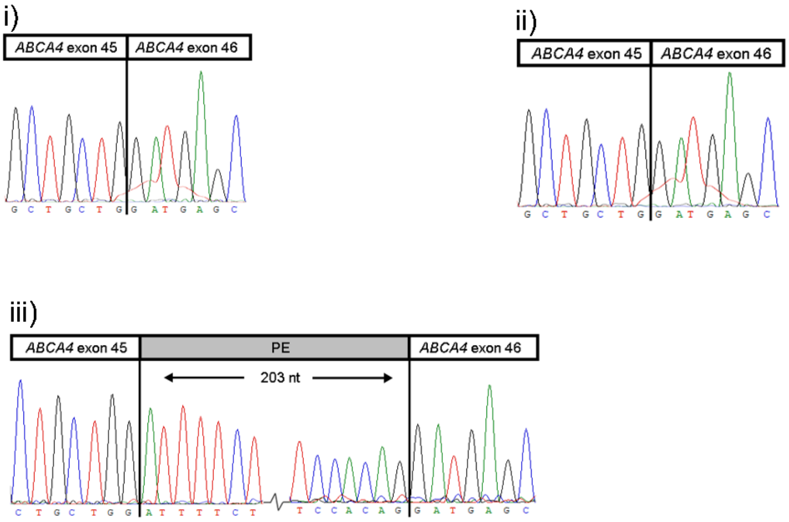

Supplementary Figure S4. Sequence analysis of the resulting RT-PCR products from rescue experiments of deep-intronic variants causing similar pseudoexon (PE) inclusion in the same region. Sanger sequencing chromatograms from splicing correction experiments targeting c.859-640A>G/c.859-546G>A (**A**) and c.4539+2064C>T/c.4539+2065C>G (**B**) variants. For each case, sequences of the correct transcript from wild-type *ABCA4* midigene condition (**i**), AON-corrected transcript in mutant *ABCA4* midigene condition (**ii-iii**) and PE inclusion transcript in mutant *ABCA4* midigene condition (**iv-vi**) are shown.

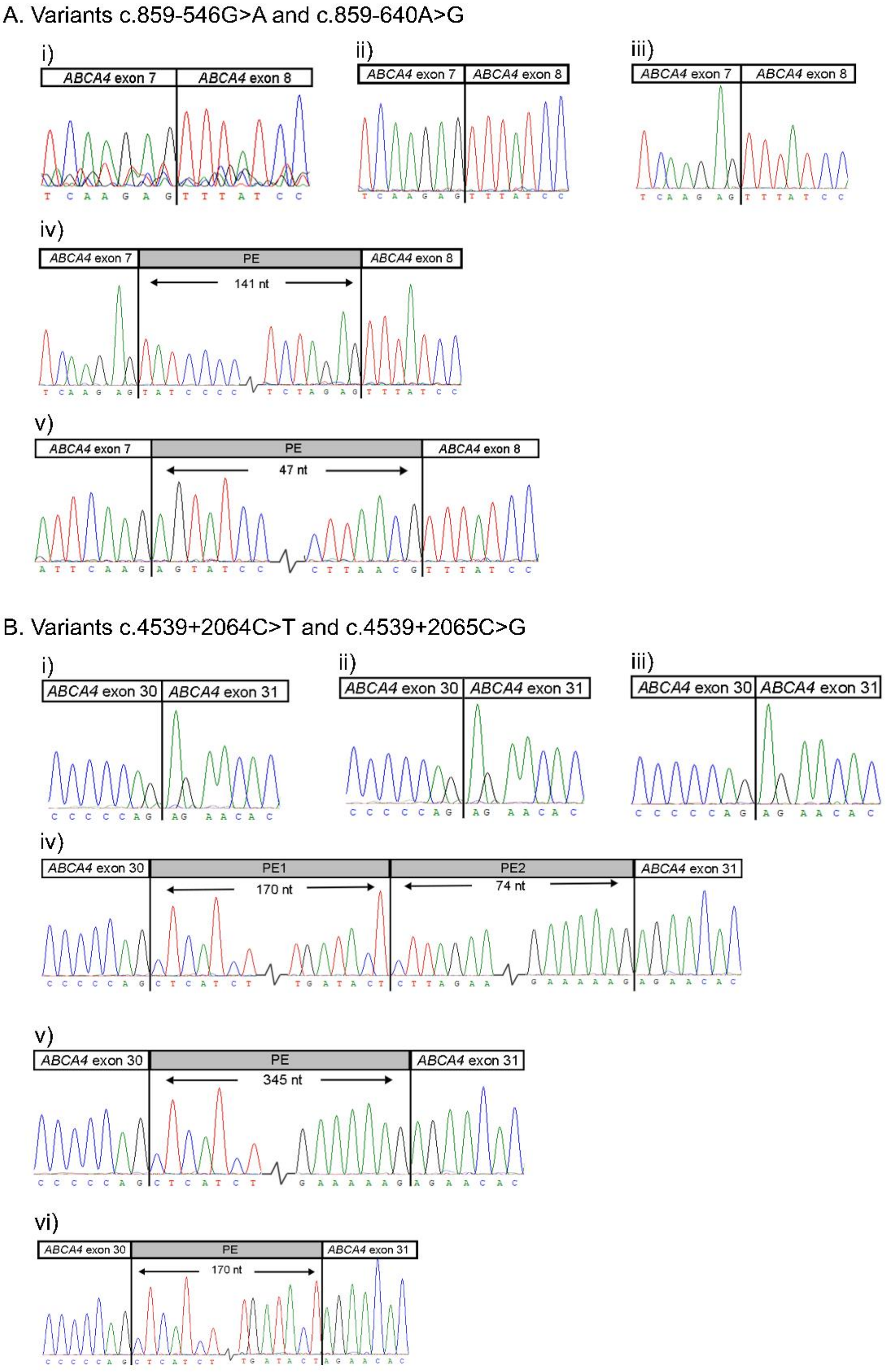

Supplementary Figure S5. Aberrant splicing rescue for c.4539+2064C>T and c.4539+2065C>G deep-intronic variants by 2'-OMe-PS AON4 and AON17. A) Analysis of splicing correction by reverse transcription polymerase chain reaction (RT-PCR) upon AON delivery. Wild-type (WT) midigenes and the corresponding mutant midigenes containing variants c.4539+2064C>T and c.4539+2065C>G were transfected in HEK293T, leaving one empty as endogenous HEK293T expression control (END). The different AONs and a SON (2'-OMe-PS) were then delivered, except for the non-treated lanes (NT). Genomic region of the different midigenes is depicted on top of each representative RT-PCR image, whereas the graphs next to each panel represent the semi-quantification of the resulting RT-PCR products, showing the % of correct and pseudoexon (PE) inclusion transcripts. MQ shows the negative control of the PCR reaction and amplification of exon 5 of Rhodopsin (RHO) gene was used as a transfection control. Data (n=2) are presented as mean±SD (*p<0.05, **p<0.01, ***p<0.001, ****p<0.0001). **B)** Predicted binding of AON17 on target pre-mRNA presenting c.4539+2064C>T or c.4539+2065C>G variants (predictions were obtained using RNA structure software: http://rna.urmc.rochester.edu/RNAstructureWeb/).

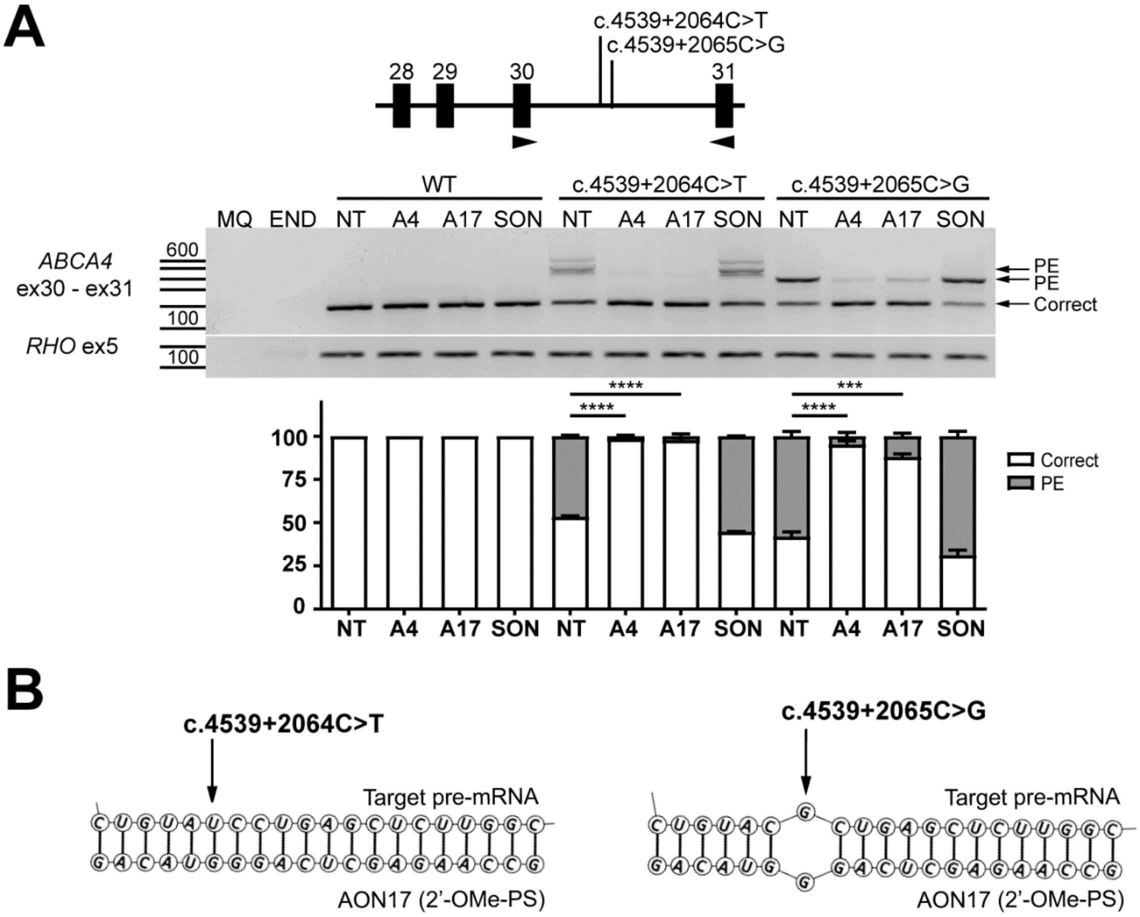

Supplementary Table S1. *ABCA4* allele count and RNA defect associated to the analyzed intronic variants (adapted from Cremers *et al.*, PRER, 2020).

Variant	Allele count	RNA defect
c.1937+37C>G	2	r.1937_1938ins1938+1_1938+36
c.3191-11T>A	1	r.3190_3191ins3191-1_3191-9
c.4352+61G>A	2	r.[4352_4353ins4352+1_4352+57,=]
c.67-2023T>G	4	r.[66_67ins67-2266_67–2024,=]
c.570+1798A>G	3	r.570_571ins570+1733_570+1797
c.769-788A>T	1	r.768_769ins769-778_769-617
c.859-640A>G	2	r.858_859ins859-685_859-640
c.859-546G>A	1	r.[858_859ins859-545_859–685,=]
c.2588-706C>T	1	r.[2587_2588ins2588-839_2588–708,=]
c.2919-826T>A	2	r.[2918_2919ins2919-957_2919–825,=]
c.3050+370C>T	2	r.3050_3051ins3050+164_3050+368
c.4539+2064C>T	27	r.[4539_4540ins4539+1891_4540–2162,=]
c.4539+2065C>G	1	r.[4539_4540ins4539+1891_4539+2060,=]
c.4634+741A>G	1	r.[4634_46354ins4634+614_4634+740,=]
c.6283-78G>T	2	r.[=,6283_6283ins6283-282_6283–80]

Supplementary Table S2. Primer sequences for site-directed mutagenesis in entry clones.

Wild-type fragment	DNA variant_primer orientation	Sequence (5’-3’)
**BA16**	c.3191-11T>A_forward	CTTAACTCTCCCGTCCTTCTAGTGCCTCCAGGTG
**BA16**	c.3191-11T>A_reverse	CACCTGGAGGCACTAGAAGGACGGGAGAGTTAAG

Supplementary Table S3. Primer sequences for validation of genomic *ABCA4* regions in entry clones.

BA_ID	Primer number	Sequence (5’-3’)	Intron/Exon	Forward/Reverse	Genomic position (hg19)	cDNA position (hg19)
**BA16**	61730	AGCAGGGTCTGCTATTTTTGT	Intron 20	Forward	94509189	3051-158
**BA16**	48620	TTGGCAATCAAGATGCTCAC	Intron 20	Reverse	94509126	3051-95
**BA16**	47862	AGATGGAAGCCATGTTGGAG	Exon 21	Forward	94508960	3122
**BA16**	61731	ACTTCACAGAGGTGTTCCCAC	Intron 21	Reverse	94508770	3190+122
**BA16**	43375	AATCACCACCTTGGCATCTC	Exon 22	Reverse	940508393	3252
**BA16**	47864	TGCGCAAGATGAAAAACATC	Exon 23	Forward	94506808	3479
**BA16**	61733	CTTTGGGAATGAAGGAAGAAG	Intron 24	Reverse	94505416	3607+183

Supplementary Table S4. Primer sequences for reverse transcription-PCR analysis.

Variant ID	Primer number	Primer sequence (5'-3')	Genomic position (hg19)	cDNA position (hg19)	Product size (bp)
**BA11 c.1937+37C>G_Forward**	47856	GCCTATCTGCAGGACATGGT	94528245	1825	218
**BA11 c.1937+37C>G_Reverse**	47855	CGCAACTCCTTCTCCAAGAC	94526211	2042	
**BA20 c.4352+61G>A_Forward**	73988	GCAGTTCACGGTACTTGCAG	94496061	4275	149
**BA20 c.4352+61G>A_Reverse**	47869	GGAACAGCTGGGTGATGTTT	94495117	4423	
**BA16 c.3191-11T>A_Forward**	47862	AGATGGAAGCCATGTTGGAG	94508960	3122	185
**BA16 c.3191-11T>A_Reverse**	71433	ATCCCAGATTGAGCGTCTC	94508339	3306	
**BA3 c.570+1798A>G_Forward**	66967	GGAATACGAATAAGGGATATCTTG	94568693	448	298
**BA3 c.570+1798A>G_Reverse**	75865	CCACGTTGGCATACAGAGTG	94564373	745	
**BA21 c.4634+741A>G_Forward**	74420	AAGACCTGACGGACAGGAAC	94490576	4568	94
**BA21 c.4634+741A>G_Reverse**	47871	TCATTGACCCAGAATTTGCTC	94488948	4661	
**BA28 c.6283-78G>T_Forward**	47884	GACTGTCTACGCCGACTGC	94467516	6180	189
**BA28 c.6283-78G>T_Reverse**	47883	ACAGCCCTCCCTTCTCTGAT	94466576	6368	
**BA13 c.2588-706C>T_Forward**	42995	TGTCCATGCAGATGATGCTC	94520741	2513	100
**BA13 c.2588-706C>T_Reverse**	47859	CAAGGAAGTGGGGTTCCATA	94517230	2612	
**BA6 c.769-788A>T_Forward**	29999	CGGAGGTCAACAACGAGTCT	129251150	587	134
**BA6 c.769-788A>T_Reverse**	47845	ACGGCTGTCTAGGAGTGTGG	94548974	792	
**BA15 c.2919-826T>A_Forward**	75935	TCTTTGAACGTGAGCATCCA	94512643	917	295
**BA15 c.2919-826T>A_Reverse**	76319	AACAGGATGTTGTGCTGTGG	94510175	3044	
**BA35 c.67-2023T>G_Forward**	29999	CGGAGGTCAACAACGAGTCT	129251150	587	274
**BA35 c.67-2023T>G_Reverse**	30000	AGGTGTAGGGGATGGGAGAC	129252614	*53	
**BA15 c.3050+370C>T_Forward**	75372	GGGACATTGAAACCAGCCTG	94510244	2975	253
**BA15 c.3050+370C>T_Reverse**	71431	TTCATTCCGCTTGTGGTG	94508911	3171	
**BA7 c.859-640A>G_Forward**	47848	TCTGAGATCTTGGGGAGGAA	94548959	807	254
**BA7 c.859-640A>G_Reverse**	47847	TGGAGTCAATCCCCAGAAAG	94546073	1060	
**BA7 c.859-546G>A_Forward**	47848	TCTGAGATCTTGGGGAGGAA	94548959	807	254
**BA7 c.859-546G>A_Reverse**	47847	TGGAGTCAATCCCCAGAAAG	94546073	1060	
**BA20 c.4539+2064C>T_Forward**	47870	AAACATCACCCAGCTGTTCC	94495136	4404	186
**BA20 c.4539+2064C>T_Reverse**	43377	ATGTTCCTGTCCGTCAGGTC	94490555	4589	
**BA20 c.4539+2065C>G_Forward**	47870	AAACATCACCCAGCTGTTCC	94495136	4404	186
**BA20 c.4539+2065C>G_Reverse**	43377	ATGTTCCTGTCCGTCAGGTC	94490555	4589	

Supplementary Table S5. Semi-quantification analysis of the RT-PCR products from non-treated (NT) or AON/SON-treated samples. The amount of the different transcripts is represented as a percent (%) of the total transcript for each condition or lane. The average of two independent experiments, followed by two independent semi-quantification for each replicate is provided. SD: standard deviation.

Condition	WT								c.1937+37C>G							
	NT		AON1		AON2		SON		NT		AON1		AON2		SON	
	%	SD	%	SD	%	SD	%	SD	%	SD	%	SD	%	SD	%	SD
Correct	100,000	0,000	100,000	0,000	100,000	0,000	100,000	0,000	0,000	0,000	100,000	0,000	84,635	0,785	0,000	0,000
Elongation	0,000	0,000	0,000	0,000	0,000	0,000	0,000	0,000	100,000	0,000	0,000	0,000	15,365	0,785	100,000	0,000

Condition	WT						c.3191-11T>A					
	NT		AON1		SON		NT		AON1		SON	
	%	SD	%	SD	%	SD	%	SD	%	SD	%	SD
Correct	100,000	0,000	100,000	0,000	100,000	0,000	0,000	0,000	0,000	0,000	0,000	0,000
Elongation	0,000	0,000	0,000	0,000	0,000	0,000	100,000	0,000	100,000	0,000	100,000	0,000

Condition	WT								c.4352+61G>A							
	NT		AON1		AON2		SON		NT		AON1		AON2		SON	
	%	SD	%	SD	%	SD	%	SD	%	SD	%	SD	%	SD	%	SD
Correct	100,000	0,000	100,000	0,000	100,000	0,000	100,000	0,000	0,000	0,000	28,400	0,509	67,070	8,316	0,000	0,000
Elongation	0,000	0,000	0,000	0,000	0,000	0,000	0,000	0,000	100,000	0,000	71,600	0,509	32,930	8,316	100,000	0,000

Condition	WT								c.67-2023T>G							
	NT		AON1		AON2		SON		NT		AON1		AON2		SON	
	%	SD	%	SD	%	SD	%	SD	%	SD	%	SD	%	SD	%	SD
Correct	90,730	5,020	76,490	12,912	83,940	9,023	88,505	8,577	26,825	5,890	35,435	0,304	90,375	13,244	21,560	14,368
PE1	9,270	5,020	23,510	12,912	16,060	9,023	11,495	8,577	56,530	13,364	13,265	11,109	0,000	0,000	64,710	18,979
PE2	0,000	0,000	0,000	0,000	0,000	0,000	0,000	0,000	16,645	7,474	51,295	11,406	9,625	13,244	13,730	4,610

Condition	WT								c.570+1798T>G							
	NT		AON1		AON2		SON		NT		AON1		AON2		SON	
	%	SD	%	SD	%	SD	%	SD	%	SD	%	SD	%	SD	%	SD
Correct	100,000	0,000	100,000	0,000	100,000	0,000	100,000	0,000	0,000	0,000	0,000	0,000	0,000	0,000	0,000	0,000
PE	0,000	0,000	0,000	0,000	0,000	0,000	0,000	0,000	100,000	0,000	100,000	0,000	100,000	0,000	100,000	0,000

Condition	WT								c.769-788A>T							
	NT		AON1		AON2		SON		NT		AON1		AON2		SON	
	%	SD	%	SD	%	SD	%	SD	%	SD	%	SD	%	SD	%	SD
Correct	100,000	0,000	100,000	0,000	100,000	0,000	97,975	2,864	20,095	4,264	94,110	2,843	94,630	4,752	15,300	0,792
PE	0,000	0,000	0,000	0,000	0,000	0,000	2,025	2,864	79,905	4,264	5,890	2,843	5,370	4,752	84,700	0,792

Condition	WT									
	NT		AON1		AON2		AON3		SON	
	%	SD	%	SD	%	SD	%	SD	%	SD
Correct	100,000	0,000	100,000	0,000	100,000	0,000	100,000	0,000	100,000	0,000
PE	0,000	0,000	0,000	0,000	0,000	0,000	0,000	0,000	0,000	0,000

Condition	c.859-640A>G									
	NT		AON1		AON2		AON3		SON	
	%	SD	%	SD	%	SD	%	SD	%	SD
Correct	0,000	0,000	100,000	0,000	100,000	0,000	24,335	5,848	0,000	0,000
PE	100,000	0,000	0,000	0,000	0,000	0,000	75,665	5,848	100,000	0,000

Condition	c.859-546G>A									
	NT		AON1		AON2		AON3		SON	
	%	SD	%	SD	%	SD	%	SD	%	SD
Correct	0,000	0,000	100,000	0,000	100,000	0,000	100,000	0,000	20,665	0,700
PE	100,000	0,000	0,000	0,000	0,000	0,000	0,000	0,000	79,335	0,700

Condition	WT								c.2588-706C>T							
	NT		AON1		AON2		SON		NT		AON1		AON2		SON	
	%	SD	%	SD	%	SD	%	SD	%	SD	%	SD	%	SD	%	SD
Correct	100,000	0,000	100,000	0,000	100,000	0,000	100,000	0,000	4,520	6,392	94,390	7,934	100,000	0,000	3,060	4,327
PE	0,000	0,000	0,000	0,000	0,000	0,000	0,000	0,000	95,480	6,392	5,610	7,934	0,000	0,000	96,940	4,327

Condition	WT								c.2919-826T>A							
	NT		AON1		AON2		SON		NT		AON1		AON2		SON	
	%	SD	%	SD	%	SD	%	SD	%	SD	%	SD	%	SD	%	SD
Correct	100,000	0,000	100,000	0,000	100,000	0,000	100,000	0,000	46,820	3,111	72,020	0,750	100,000	0,000	42,335	1,690
PE	0,000	0,000	0,000	0,000	0,000	0,000	0,000	0,000	53,180	3,111	27,980	0,750	0,000	0,000	57,665	1,690

Condition	WT								c.3050+370							
	NT		AON1		AON2		SON		NT		AON1		AON2		SON	
	%	SD	%	SD	%	SD	%	SD	%	SD	%	SD	%	SD	%	SD
Correct	100,000	0,000	100,000	0,000	100,000	0,000	100,000	0,000	0,000	0,000	61,275	2,595	71,070	9,065	0,000	0,000
PE1	0,000	0,000	0,000	0,000	0,000	0,000	0,000	0,000	89,855	5,480	10,015	3,345	25,035	3,557	91,155	6,979
PE2	0,000	0,000	0,000	0,000	0,000	0,000	0,000	0,000	10,145	5,480	28,715	0,742	3,895	5,508	8,845	6,979

Condition	WT							
	NT		AON4		AON17		SON	
	%	SD	%	SD	%	SD	%	SD
Correct	100,000	0,000	100,000	0,000	100,000	0,000	100,000	0,000
PE	0,000	0,000	0,000	0,000	0,000	0,000	0,000	0,000

Condition	c.4539+2064C>T							
	NT		AON4		AON17		SON	
	%	SD	%	SD	%	SD	%	SD
Correct	54,700	5,049	100,000	0,000	87,090	8,330	52,765	6,428
PE	45,300	5,049	0,000	0,000	12,910	8,330	47,235	6,428

Condition	c.4539+2065C>G							
	NT		AON4		AON17		SON	
	%	SD	%	SD	%	SD	%	SD
Correct	38,920	11,257	100,000	0,000	72,350	11,865	35,355	11,703
PE	61,080	11,257	0,000	0,000	27,650	11,865	64,645	11,703

Condition	WT								c.4634+741A>G							
	NT		AON1		AON2		SON		NT		AON1		AON2		SON	
	%	SD	%	SD	%	SD	%	SD	%	SD	%	SD	%	SD	%	SD
Correct	100,000	0,000	100,000	0,000	100,000	0,000	100,000	0,000	21,590	7,934	100,000	0,000	100,000	0,000	19,630	6,831
PE1	0,000	0,000	0,000	0,000	0,000	0,000	0,000	0,000	13,905	9,694	0,000	0,000	0,000	0,000	16,265	9,525
PE2	0,000	0,000	0,000	0,000	0,000	0,000	0,000	0,000	64,505	17,628	0,000	0,000	0,000	0,000	64,110	16,362

Condition	WT								c.6283-78G>T							
	NT		AON1		AON2		SON		NT		AON1		AON2		SON	
	%	SD	%	SD	%	SD	%	SD	%	SD	%	SD	%	SD	%	SD
Correct	100,000	0,000	100,000	0,000	100,000	0,000	100,000	0,000	70,500	1,400	100,000	0,000	100,000	0,000	68,530	3,295
PE	0,000	0,000	0,000	0,000	0,000	0,000	0,000	0,000	29,500	1,400	0,000	0,000	0,000	0,000	31,470	3,295
